# Microfluidic Systems to Mimic the Blood–Brain Barrier: from Market to Engineering Challenges and Perspectives

**DOI:** 10.1021/acsbiomaterials.4c02221

**Published:** 2025-06-25

**Authors:** Gabriela Gomes da Silva, Daniel Pereira Sacomani, Bruna Gregatti de Carvalho, Marimélia Aparecida Porcionatto, Angelo Gobbi, Renato Sousa Lima, Lucimara Gaziola de la Torre

**Affiliations:** † Department of Material and Bioprocess Engineering, School of Chemical Engineering, University of Campinas (UNICAMP), Campinas, São Paulo 13083-970, Brazil; ‡ National Institute of Science and Technology in Modeling Human Complex Diseases with 3D Platforms (INCT Model3D), São Paulo, São Paulo 04039-032, Brazil; § Department of Biochemistry, Escola Paulista de Medicina, Universidade Federal de São Paulo, São Paulo 04039-032, Brazil; ∥ Brazilian Nanotechnology National Laboratory, Brazilian Center for Research in Energy and Materials, Campinas, São Paulo 13083-970, Brazil; ⊥ Institute of Chemistry, University of Campinas, Campinas, São Paulo 13083-970, Brazil; # Center for Natural and Human Sciences, Federal University of ABC, Santo André, São Paulo 09210-580, Brazil; ¶ São Carlos Institute of Chemistry, University of São Paulo, São Carlos, São Paulo 13565-590, Brazil

**Keywords:** blood–brain barrier, microfluidics, organ-on-a-chip, BBB-on-a-chip

## Abstract

Studying and understanding complex biological systems is a challenge that requires technologies that go beyond traditional cell culture methods. Among the new technologies that have been developed in recent times, blood–brain barrier-on-a-chip (BBB-on-a-chip) models are becoming popular. Due to their ability to integrate fluid flow, which is absent in traditional static models, it has been possible to create a cellular microenvironment that mimics blood vessels and blood flow. In addition, the possibility of coculturing different cell types in multicellular models allows the observation of their interactions and increases interest in these systems. With different possibilities in terms of prototyping techniques (e.g., laminate manufacturing, molding, and 3D impression), chip designs (e.g., planar and cylindrical configurations), and materials (e.g., thermoplastics, elastomers, and hydrogels), the number of publications in the BBB research field has significantly increased in the last five years. In parallel, the emergence and consolidation of several companies have made the commercialization and application of these chips possible, mainly in the pharmaceutical area, which is not yet integrated into the drug development pipeline. In this context, the present review describes the intersection between technique, market, and applications that mimic the BBB. We showed organ-on-a-chip (OoC) market growth and the collaborative research between the main OoC supplier companies and industrial collaborators. Also, we present an overview of the primary fabrication methods used in constructing the OoC systems and their application in developing the BBB models. In addition, we discussed the BBB-on-a-chip designs developed in the last five years, including their engineering aspects (such as materials, dimensions, and configuration), characterization, and challenges in mimicking the BBB.

## Graphical abstract

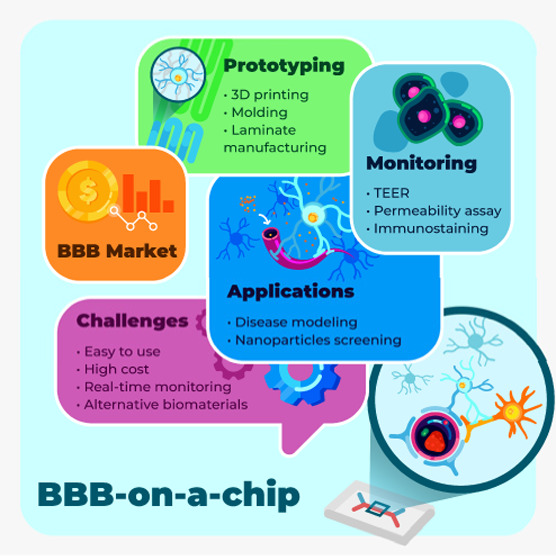

## 1 Introduction

In the last decades, the scientific community and pharmaceutical industry have faced challenges in developing new drugs and delivery nanoformulations, also known as vectors, that can effectively treat specific brain diseases such as stroke, Huntington’s, Parkinson’s, Alzheimer’s, or Lou Gehrig’s diseases.^1,2^ This demand is increasing due to the aging population, with a projected 2 billion elderly by 2050 according to WHO, which increases the risk of developing neurodegenerative diseases like Alzheimer’s and Parkinson’s, the most common neurodegenerative diseases, affecting 5 and 1% of the population over 60 years of age, respectively.^3–7^ One of the significant challenges in developing novel drugs for preventing and treating these neurodegenerative diseases is finding an efficient vector for brain delivery (i.e., promising candidates for targeted delivery owing to their biocompatibility and biodegradability, such as biomimetic nanoparticles and liposomes) that can access the neurovascular unit (NVU) and efficiently cross the blood–brain interface, known as the blood–brain barrier (BBB).^8–10^

In the body, NVU is a complex structure composed of brain microvessel endothelial cells (BMECs), pericytes (PRCs), astrocytes (ACs), neurons, oligodendrocytes, and microglia (Figure 1), which is associated with local microcirculation and metabolism.^11^ The BBB is part of the NVU and is responsible for regulating the transport of any molecules from the blood to the brain, providing selective protection to the brain.^12,13^ The BBB (Figure 1) is a multicellular complex structure composed of BMECs that line the inner surface of cerebral blood vessels, PRCs wrapping around BMECs, and ACs that contact blood vessels and neurons.^12,14^ The BBB also involves other essential components, such as neurons, microglia, and extracellular matrix (ECM), forming the NVU. One of the most important cell types in the BBB, BMECs have specialized tight junctions (TJs) and transport mechanisms that carefully control the transfer of nutrients and ions through the BBB while working to protect the brain from pathogens and harmful toxins.^14–16^ However, while this selectivity is essential, it can complicate the entry of therapeutic drugs into the brain, posing a critical challenge for drug development.^17^

**Figure 1:**
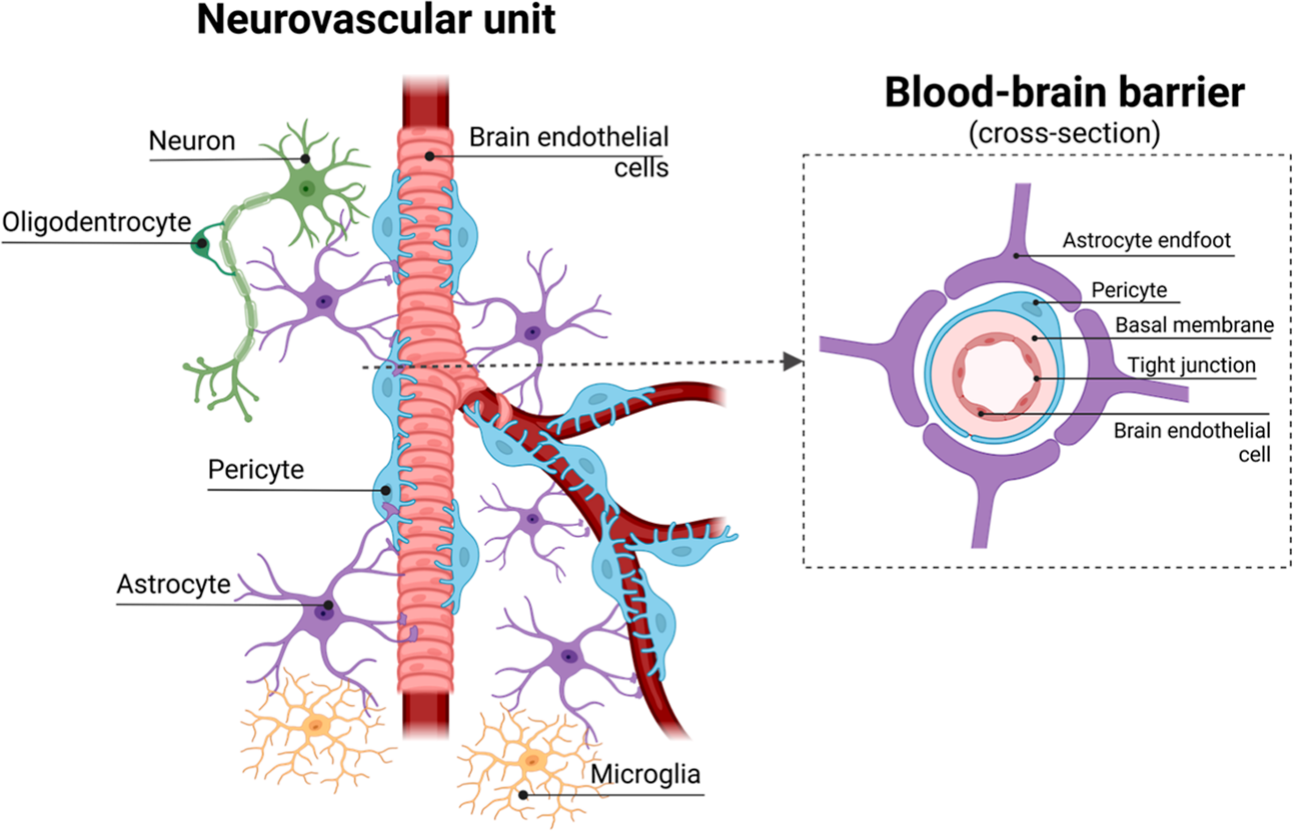
Components of NVU comprising the BBB and adjacent cells (created with Biorender.com).

Preclinical tests are one of the steps in drug development in which the efficacy of drug candidates is tested using in vitro and in vivo assays. In this sense, brain drugs are usually tested in two-dimensional (2D) in vitro models (i.e., Transwell models), which are useful for drug testing and for studying the biology of cerebral ECs.^1^ However, they do not fully replicate the complex interactions of the NVU.^1,18^ Another challenge in 2D in vitro models is precisely controlling fluid flow at the BBB, which should generate shear stress ranging from 5 to 23 dyn cm^–2^, crucial for maintaining barrier functions, regulating transport, and controlling physiological functions in the brain parenchyma.^14^ In static conditions, the conventional in vitro cell culture models fail to mimic the fluid flow, an important parameter to ensure adequate cell shear stress.^13^ In contrast, in vivo animal models are very complex, but they do not always mimic human physiology or disease, and establishing these models is expensive and laborious and involves ethical concerns.^19,20^

Furthermore, there is growing evidence that the function of the BBB is linked not only to the treatment of brain diseases (such as Alzheimer’s disease, Parkinson’s disease, Huntington’s disease, brain cancer, etc.) but also to their progression.^21^ When the integrity of the BBB is compromised, it becomes more permeable, and the transport to the brain becomes unbalanced, leading to the infiltration of harmful molecules and toxins into the NVU, which may accelerate neurodegeneration and contribute to the onset of neurological disorders.^16,21,22^ The challenges in studying drug delivery also apply to brain diseases. Traditional 2D models, while more accessible when compared with animal models, do not effectively replicate the intricate 3D physiology of the BBB.^23^

To overcome the limitations of traditional in vitro methods, microphysiological systems called organs-on-a-chip (OoCs) have been used. OoCs are microfluidic systems that allow the flow under small flow rates within microscale channels, thus enabling the growth of cells in perfusion systems.^24^ These systems consist of miniature tissues growing in dynamic microfluidic chips.^25^ One advantage of adopting OoCs is that they can recapitulate one or more tissue-specific functions, culturing different cell types, especially those that require fluid shear stress (e.g., vascular and lymphatic ECs, or epithelial cells of the kidney and lung). Another advantage is that OoCs require small volumes of reagents when compared with traditional in vitro methods, which are usually expensive (such as cell culture media, drugs, etc.).^25,26^ In this sense, the OoC technology can offer a more physiologically relevant culture environment for BBB modeling than 2D in vitro models. BBB-on-a-chip can allow a comprehensive understanding of the BBB’s protection mechanism and brain diseases, providing insights into improving the delivery of drugs and nanomedicines across the barrier.

From a manufacturing and design standpoint, the BBB-OoC devices used to study the health of the BBB, drug delivery, or brain diseases are fundamentally the same. The key difference between these conditions lies in the types of cells involved. This can include the use of cells harboring mutations,^27–29^ the exposure of cells to specific molecules,^30–32^ or the presence of other cell types.^33,34^ Also, it is crucial to distinguish between BBB-on-a-chip and brain-on-a-chip models. Many models referred to in the literature as BBB often lack the essential components required for barrier formation, such as BMECs. As a result, these models can be more accurately classified as brain models, particularly when focusing on neuron cultures. Additionally, various disease models have been examined in numerous studies, including neuron cultures exposed to conditions associated with specific diseases.

The scientific community has made significant progress in developing microphysiological systems to study the BBB-OoC, with research in these models advancing rapidly over the last five years. According to Figure 2A, there was an increase in the number of publications related to the OoC over the years, similar to the significant rise in BBB publications observed after 2020. These papers have a multidisciplinary range of knowledge areas (Figure 2B) involving, e.g., biochemistry and molecular biology, chemistry, engineering, neuroscience and neurology, and science and technology. Also, several startups have been growing and contributing to further yield advances in the OoC field that can potentially impact BBB-related studies.

**Figure 2:**
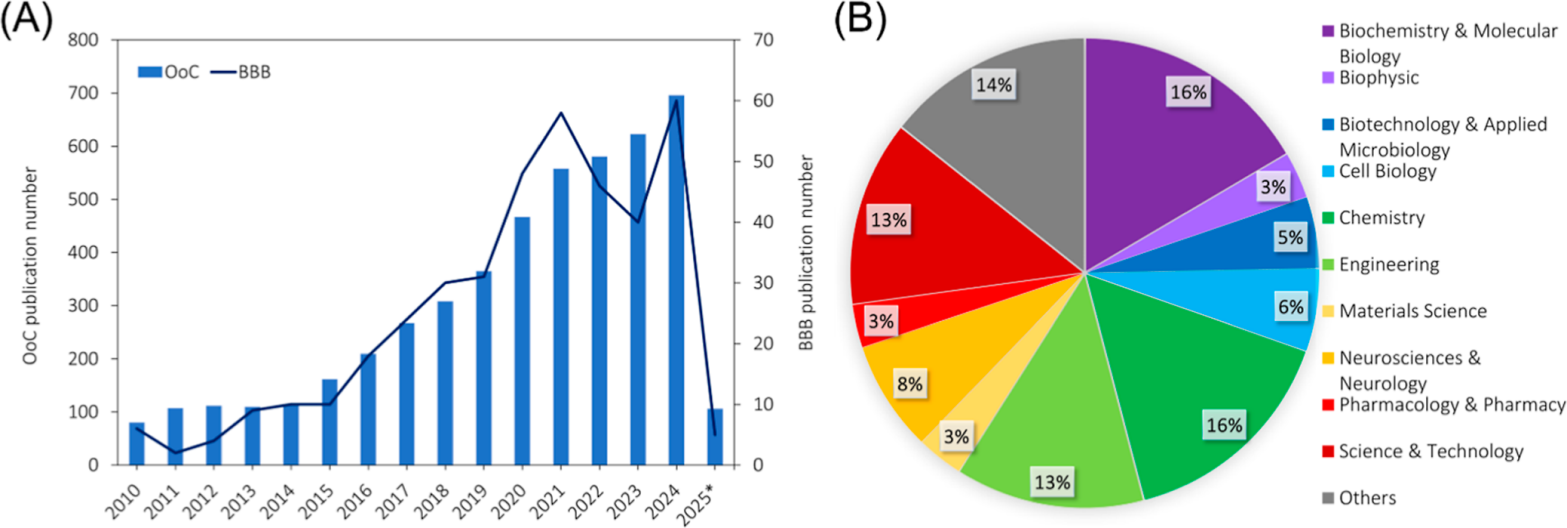
Graphical report of the cumulative number of Web of Science (WoS) Analytic Reports publications.^35^ (A) Report of publications about the OoC and BBB-on-chip over the last years (2010–2025*, *up to February). (B) Percentage of articles published in the area of knowledge. Keywords used for the WoS database search: OoC, organ-on-a-chip, organ-on-chip, organ on a chip, microphysiological system, BBB-on-a-chip, blood–brain barrier chip, BBB microfluidic device, and BBB microfluidic device.

In this sense, this review compiles the research reported in the literature over the last five years (from 2020 to February 2025), focusing on BBB-on-a-chip models, their engineering aspects, and the challenges of monitoring and characterizing them. Despite that, we provide an extensive market review involving the emergence and growth of startups, the commercialization of chips and accessories for operation, investments received by these companies, and collaborative research involving their use with industrial collaborators. In short, we present the available methods for BBB construction, research trends, and the market from OoC to BBB in an integrated manner, highlighting the inherent challenges that research in the BBB field faces.

## 2 From OOC to BBB Market

Microfluidic systems have been used in biological research for over 20 years, and the development of the first microfluidic devices started with soft lithography and polydimethylsiloxane (PDMS). Since 2010, attractive OoC systems have been developed by combining cell biology and microsystem technologies using PDMS and other materials.^36,37^ Several startups have emerged to develop OoC technologies that are not yet integrated into the drug development pipeline. These companies use unique tissue assembly methods to produce lab-scale prototypes and occupy this market space.^38^ Table 1 gives an overview of these companies from various sources, including Google Scholar, PubMed, and other publications.^37,39,40^ The projected global market size for OoC is expected to increase from US$41 million to US $303.6 million by 2026, with an average annual growth rate ranging from 38% to 57%.^41,42^ During the projected period (by 2026), North America is expected to keep the largest share of the OoC market, where 42% of the OoC startups are currently localized in the USA and Canada, as shown in Figure 3. The USA’s dominant position in the OoC industry (as shown in Figure 3 and Table 1, with 39% of OoC companies) can be attributed to favorable government initiatives that provide funding and programs for essential drug development projects in research groups and pharmaceutical companies.^41^

**Table 1:** Timeline of OoCs Companies Worldwide, Based on Research on Google Scholar^43^ and PubMed^44^

foundation year	company	applications	country
2006	HuREL	liver chip	USA
2007	Hepregen	liver, islet, cancer model, accessory devices	USA
2008	Xona microfluidics	brain, neuron chips	USA
2012	Nortis BIO	kidney, liver, multi-organ-on-a-chip, accessory devices	USA
2013	Emulate Inc.	liver, kidney, lung, intestine, brain chips, accessory devices	USA
2014	AxoSim	nerve-on-a-chip	USA
2014	TARA Biosystems	Biowire II platform	USA
2014	SynVIVO	neuroscience, toxicology, inflammation, and oncology	USA
2015	Hesperos	heart, liver, lung, brain, skin, kidney chips, multiorgan-on-a-chip	USA
2016	Altis BioSystems	RepliGut Kits	USA
2019	Aracari Bio	vascularized micro-organ chip	USA
2019	Draper	lung, liver chips	USA
2001	Ibidi GmbH	brain, neurons, lung, liver, gut, kidney, islet, cartilage, microvasculature, skin chips	Germany
2010	TissUse	multi-organ-on-a-chip accessory devices	Germany
2015	EHT Technologies	heart-on-a-chip	Germany
2018	Dynamic42	lung, liver, gut chips	Germany
2013	Mimetas	kidney, gut, tumors, liver, lung, intestine, blood vessel, neuronal models, accessory devices	The Netherlands
2017	BI/OND	organoid cultivation, tissue–tissue interface	The Netherlands
2009	InSphero	liver, islet, tumor cell culture	Switzerland
2015	AlveoliX	lung-on-a-chip	Switzerland
2012	Aim biotech	brain, neurons, islet, cartilage, microvasculature chips	Singapore
2019	REVIVO Biosystems	skin-on-a-chip	Singapore
2014	MicroBrainBT	brain, neuron chip	France
2016	MesoBioTech	microfluidics, lung chips	France
2006	Kirkstall	Quasi Vivo system	UK
2009	CNBio	liver, gut, skin, heart, lung, kidney, brain chips	UK
2015	Ananda Devices	neuro device	Canada
2018	DAXIANG	liver and cancer chips	China
2016	BEOnChip	gut-on-a-chip	Spain
2017	Biomimx	heart-on-a-chip	Italy
2017	Jiksak Bioengineering	nerve organoids	Japan

**Figure 3:**
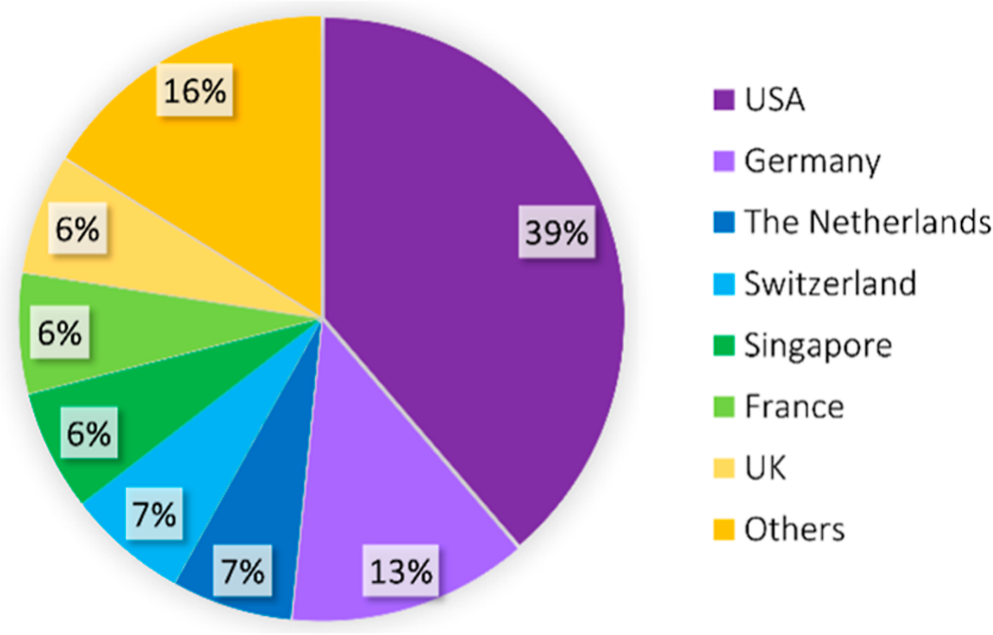
Representative diagram showing the main countries in the world where OoC companies are identified based on research from Google Scholar^43^ and PubMed.^44^ The percentage indicates the number of OoC companies in the countries (USA, Switzerland, UK, Singapore, France, Germany, The Netherlands, and others).

According to Table 1, the single-organ models most widely investigated by OoCs companies of the OoCs are the liver, kidney, heart, intestine/gut, lung, blood vessels, and brain. There are also some multicellular platforms, such as OrganoPlate from Mimetas (The Netherlands), which presents microfluidic 3D cell cultures in a pump-free perfusion system, and HUMIMIC from TissUse (Germany), a multiorgan platform with a micropump on-chip. Some companies are focused just on brain chips, such as Xona Microfluidics (USA), MicroBrainBT (France), and Anada Devices (Canada). So far, our search has not identified any companies that produce chips specifically for BBB. However, it is important to note that brain chips and multipurpose chips have also been reported for use in applications related to the BBB.^32,45–48^ As a result, distinguishing between the OoC and brain-OoC markets from the BBB-OoC market remains challenging. These challenges are associated with geometry, model complexity, price, and the need for more sophisticated and integrated techniques to characterize models as the BBB (as discussed in Section 4.1).

Among the mentioned companies, Mimetas, TissUse, and Emulate are the top three OoC industry suppliers, offering integrated development, sales, and services, with Roche, Johnson & Johnson, GSK, and AstraZeneca as the leading pharmaceutical customers.^49^ Mimetas, founded in Leiden, The Netherlands, in 2013, is now a multinational company with operations in Asia, Europe, and the USA.^50^ In terms of investments, in 2022, Mimetas was a partner in the Oncode Accelerator initiative, for which the Dutch National Growth Fund granted an amount of €325 M to accelerate and improve oncology drug development.^51^ Also, The Netherlands has funded a national OoC initiative (NOCI) with €18.8 M for ten years.^52^ Mimetas offers a multicellular platform featuring a three-channel design with a 3D gel region flanked by 2 channels (OrganoPlate) that can be modified to suit different cell types and configurations, allowing for replicating various organ functions. They also provide two instruments to work with OrganoPlate: OrganoTEER, an impedance-based TEER measurement, and OrganoFlow, which drives precisely controlled perfusion flow in OrganoPlate. Recently, Mimetas’s models were used in 21 collaborative publications (Table 2), of which only 3 reported works (representing about 14%) have used these chips in research with BBB, with applications focusing on transport and permeability through endothelial cells.^53–55^

**Table 2:** Collaborative Publications of the Top Three OoC Industry Suppliers (Mimetas, TissUse, and Emulate) in the Last 5 Years

Mimetas			
OoC	applications	industrial collaborators	ref
liver	analysis of reproducibility and robustness of high-throughput platform	Sanofi, Merck	^62^
kidney	renal ischemia/reperfusion injury model	Astellas Pharma	^63^
kidney	Lowe syndrome/Dent II disease model	Galapagos BV	^64^
kidney	high-throughput nephrotoxicity assessment of novel drug candidates	Pfizer, Roche, GSK	^65^
gut	drug absorption with Caco-2 tubules on chip	Astellas Pharma	^66^
gut	inflammatory bowel disease (IBD) on chip	Galapagos BV	^67^
gut	IBD model on a chip	Roche	^68^
gut	IBD model on a chip	Philip Morris International	^69^
gut	IBD model on a chip	Galapagos BV	^70^
blood-vessel	effect of cigarette smoke and heated tobacco products on atherosclerosis	Japan Tobacco Inc.	^71^
blood-vessel	angiogenesis in systemic sclerosis and drug testing	Galapagos	^72^
blood-vessel	endothelial inflammation	AstraZeneca	^73^
blood-vessel	transendothelial migration of T cells	Merck	^74^
blood-vessel	screening of antiangiogenic compounds	Ncardia	^75^
blood-vessel	impact of tobacco heating system on endothelial microvessels	Philip Morris International	^76^
blood–brain barrier	evaluate receptor-mediated transcytosis of therapeutic antibodies	UCB Biopharma	^53^
blood–brain barrier	evaluate permeability of compounds in BBB	Axcelead Drug Discovery Partners	^54^
blood–brain barrier	screening of endothelial cell barrier inducers using BBB	Roche	^55^
blood–retinal barrier	screening of leakage mediators and cytokine inhibitors of BRB	Roche	^77^
cancer	immunotherapy in a lung tumor-on-a-chip	GKS	^78^
cancer	infiltration assay with Caco-2-barrier	Roche	^79^

TissUse			
OoC	applications	industrial collaborators	ref
liver	comparison of static well plate vs dynamic chip model for drug-induced liver injury (DILI)	UCB Biopharma	^80^
skin-liver	investigate the genistein using the skin-liver model	Beiersdorf AG, Pharmacelsus GmbH	^81^
skin-liver	investigate the kinetics and first-pass skin metabolism of the hair dye	Beiersdorf AG, Pharmacelsus Gmb	^82^
skin-liver	investigate the kinetics and first-pass skin metabolism of the hair dye	Beiersdorf AG, Pharmacelsus GmbH, Pierre Fabre Dermo-Cosmetique	^83^
skin-liver	evaluate pharmacokinetics and pharmacodynamic properties	Beiersdorf AG, Pharmacelsus GmbH, Pierre Fabre Dermo-Cosmetique	^84^
liver-pancreas	type II diabetes model	AstraZeneca	^85^
skin-liver-thyroid	evaluate effect of topically applied chemicals	Beiersdorf AG, Pharmacelsus GmbH, LifeNet Health, Pierre Fabre Dermo-Cosmetique	^86^
brain-liver	investigate permeation at the blood–brain barrier	Pharmacelsus GmbH	^87^
thyroid-liver	thyroid hormones homeostasis model	Bayer	^88^
liver-lung	establish liver-lung model and evaluate aflatoxin B1 toxicity	Philip Morris International	^89^

Emulate Inc.			
OoC	applications	industrial collaborators	ref
liver	drug-induced liver injury (DILI) model	Abbvie, J&J	^90^
gut-tumor	On-target safety liability prediction in gut and lung chips	Roche	^91^
gut	inflammatory bowel disease (IBD) model	Takeda	^92^
gut	effects of human milk oligosaccharides on the adult gut microbiota and barrier function	Prodigest Bv, Glycom A/S	^93^
lung	live human rhinovirus 16 (HRV16)-induced asthma exacerbation model	Merck	^94^

TissUse, a biotech company from Berlin (2010), has developed different multi-organ platform designs known as HUMIMIC chips. HUMIMIC furnishes preclinical insights using human tissues on a systemic level and offers new approaches to predict toxicity profiles and efficacy in vitro, minimizing laboratory animal testing and optimizing human clinical trials.^56^ In addition to the HUMIMIC platforms, TissUse offers (i) HUMIMIC ActSense, combining impedance spectroscopy to measure TEER and high-resolution sensing and stimulation of electrically active tissue, and (ii) HUMIMIC HeatSupport, which maintains the platform in stable conditions—even outside of the incubator.^57^ In terms of investments, no data was found. Recently, TissUse has cooperated with Roche to develop drugs using multiorgan-on-a-chip technology.^52^ Also, HUMIMIC platforms were utilized in 10 recent collaborative studies (Table 2), with only one reporting the use of a multiorgan brain-liver platform to investigate drug permeation through the BBB.^58^

Emulate Inc. is an OoC technology-specialized company that was founded in 2013 at the Wyss Institute at Harvard University (USA) and has created an accurate representation of human biology and diseases. Emulate has partnered with major companies such as AstraZeneca, Johnson & Johnson, Merck, Takeda, Roche, and the FDA (U.S. Food and Drug Administration) to validate the effectiveness of their various products. They evaluate the safety and effectiveness of drug candidates for human use in an industrial setting.^36,41,59^ They offer two platform designs: Chip-S1, a PDMS chip with a porous membrane separating the channels, and Chip-A1, a complex 3D system incorporating a hydrogel. They also offer platforms for OoC culture: (i) POD, a portable module that houses the chip, containing media and effluent; (ii) ZOE CM2, a precise microenvironment control to adjust media flow rates and stretch parameters; (iii) ORD to monitor the performance of ZOE Culture modules; and (iv) the software to design organ-chip studies, remotely control and monitor ZOE CM2, and analyze results.^60^ In 2021, Emulate closed an $82 M Series E financing to scale amid rapid growth in the organ-on-a-chip market, and since its 2013 founding, it has received a funding total of nearly $225 M by the Series E financing round (with Northpond Ventures and Perceptive Advisors as backers).^61^ In recent years, five collaborative publications have reported using Emulate’s chips (Table 2); however, none have focused on or applied them to BBB.

Based on the collaborative studies in Table 2, the pharmaceutical industry has shown a significant interest in the OoC market. The major interests are studying the absorption, distribution, metabolism, excretion, and toxicity (ADMET) of the drugs during the discovery phase and disease modeling, emphasizing liver, kidney, and gut models. Specifically, in the BBB field, few collaborative works were reported, with a highlight on the study of permeability and transcytosis on BMECs, representing only 8% of publications. This shows that existing commercial models, even though they are multipurpose, probably still do not accurately mimic the BBB. In addition, to supply specific demands in the brain research field, mainly in neuron cell culture, some startups focus on constructing models with channels separated by microgroove barriers for this application. As an example of companies in the neuron cell culture, there is the Canadian startup Ananda Devices (Canada—2015), which is a biotech company providing innovative neuron-on-a-chip technology focused on neuroscience, oncology, and infectious diseases, with collaborators with more than 20 years of experience in neuroscience.^95^ They have already been commercialized in 14 different countries. Recently, Magdesian et al. used the commercial neuron-on-a-chip to propose a new method for fast neurite extension and functional neuronal connection, showing the intrinsic capacity of axons for elongation, including that of their cytoskeletal components.^96^ Xona Microfluidics (USA—2008) is a company focused on neuroscience, which has already distributed and supported its products directly to hundreds of research organizations in more than 20 countries. Its platforms provide compartmentalization, fluidic isolation, and improved cellular organization over traditionally chaotic neuronal cell cultures contained within the footprint of a standard microscope slide and are compatible with high-resolution, live, and fluorescence imaging.^97^ Recently, Nagendran et al. illustrated the compatibility of Xona Microfluidic chips for long-term neuronal cultures (>3 weeks).^98^

Although many of the commercial models available are multipurpose and suitable for BBB research, the simplicity in design, geometry, and characterization leads to failures in some aspects, resulting in outcomes that deviate from what is expected when compared to in vivo. As a result, the scientific community has recently delivered significant efforts to develop new, more affordable designs and microchips for studying the BBB, as evidenced by the growing number of publications in this area (Figure 2). In this sense, different manufacturing methods (i.e., laminate manufacturing, molding methods, and 3D printing) and designs (i.e., planar and cylindrical) have been explored to construct models to mimic the BBB. In the following section, the main techniques, along with their advantages and disadvantages, will be explained.

## 3 Overview of Standard Techniques to Fabricate BBB Microchips

Due to the demand for developing novel and efficient OoC and BBB chips, several microchip prototyping techniques have emerged to meet the required dimensions and designs for biomedical applications. All these techniques try to draw back the main limitation, i.e., the high cost associated with clean room management, of the conventional soft lithograph, which was introduced by Xia and Whitesides in 1998.^99^ In this context, the following section will discuss the main characteristics of these novel techniques and their use in BBB research.

### 3.1 Laminate Manufacturing

Polymer layers, including acrylic, polycarbonate, PDMS, adhesive transfer tapes, and glass slides, can be used for the development of master molds or laminate microfluidic devices, in which each layer is cut individually and bonded with many different techniques.^100^ Polymers and glass slides are common due to their optical clarity, low cost, and sample compatibility. The cutting method, using a knife plotter (xurography technique) or laser cutter^80,81^ (illustrated in Figure 4A) can significantly affect the device’s dimensions and functionality.

**Figure 4:**
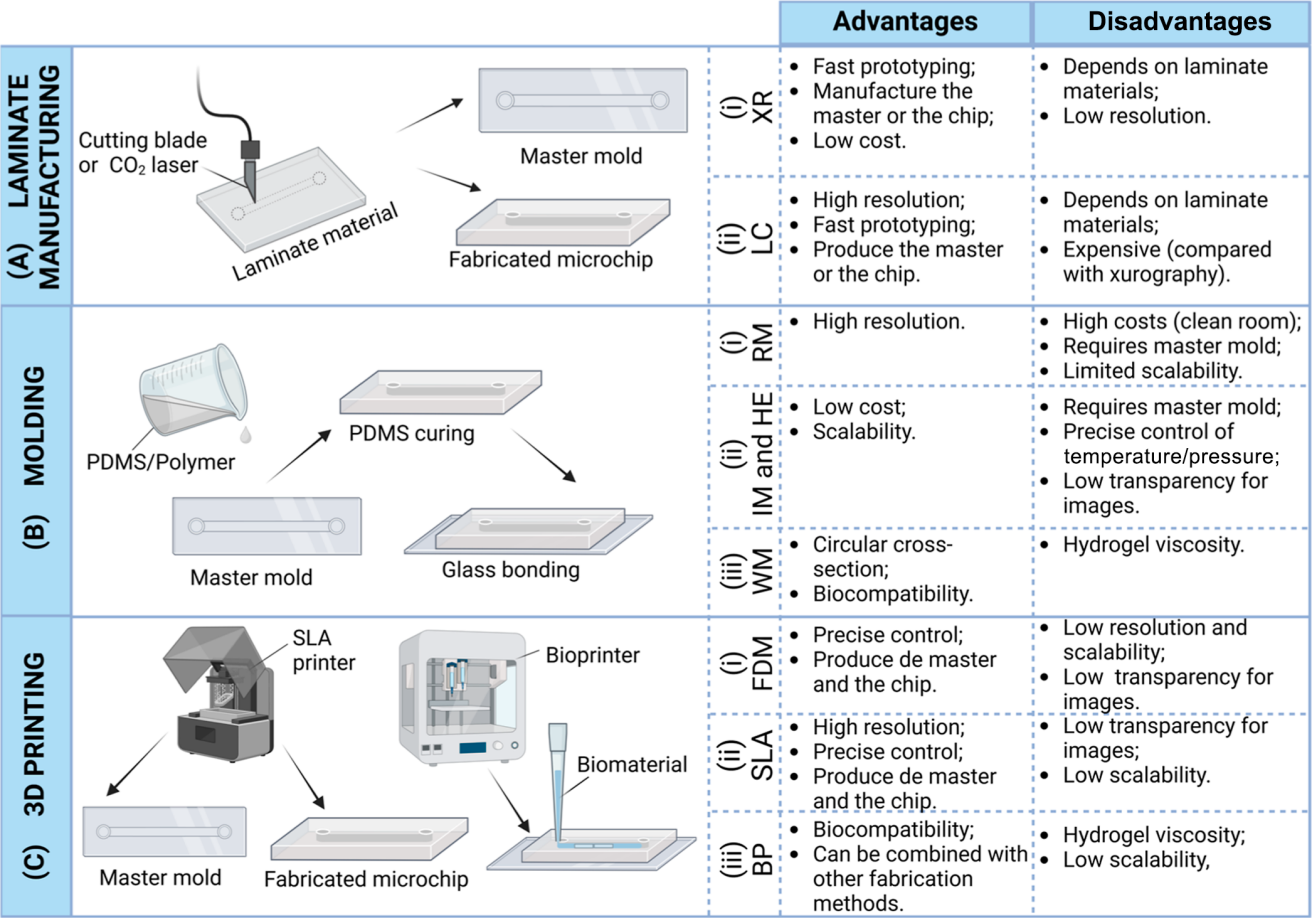
Main fabrication methods (A: laminate manufacturing, B: molding, and C: bioprinting) for BBB microchip production and their advantages and disadvantages. A­(i) XR: xurography, A­(ii) LC: laser cutting, B­(i) RM: replica molding, B­(ii) IM: injection molding and HE: hot embossing, B­(iii) WR: wire molding, C­(i) FDM: fused deposition modeling, C­(ii) SLA: stereolithography, C­(iii) BP: bioprinting (created using Biorender.com).

Xurography, Figure 4A­(i), is a low-cost, rapid, and simple microfabrication technique that does not require clean room facilities and specialized persons.^101^ A cutting plotter machine is used to remove the surplus materials from polymer films to fabricate microchannels or from adhesive vinyl films, creating masks and master molds.^101,102^ Compared to other conventional fabrication methods, xurography is a fast-prototyping technique.^103^ The thickness of the material defines the height of channels, and achieving small dimensions is challenging due to the poor resolution for sizes smaller than 500 μm^104^ and to difficulties in manipulation and alignment.^105^

On the other hand, laser cutting, Figure 4A­(ii), is a process that involves the use of laser energy to interact with material and cut it with precision, using a focused beam (traditionally CO_2_ lasers) to remove materials from the sheet surface, patterning the final microchannel or master mold.^106^ Because of the localized heat gradient caused by a long pulse laser, a maximum resolution of 50 μm could be obtained through the CO_2_ laser. Short-pulsed lasers can further augment this resolution to 10–20 μm.^101^ Laser cutting is more expensive than xurography, but when compared to methods that require cleanroom facilities, it has been considered a more accessible fabrication technique.^107^

In general, techniques involving laminate manufacturing to construct the OoC and BBB-OoC have been rarely reported. The last report was in 2022, in which a macro device was fabricated using a poly­(methyl methacrylate) (PMMA) mold obtained by laser cutting.^108^ They developed a microfluidic model of vascularized glioblastoma, in which a tumor spheroid was in direct contact with self-assembled vascular networks composed of human ECs, ACs, and PRCs. Paoli and collaborators also used laminate manufacturing to propose a rapid prototyping device by combining laser cutting and xurography. They developed a microfluidic prototype with thermoplastic sheets (Cyclic Olefin Copolymer (COC) and PMMA) to mimic biological barriers and their permeability. Brain ECs and PRCs were used to construct a simplified BBB and to validate the model, showing excellent optical characteristics and biocompatibility.^109^

### 3.2 Molding

Another technique used for microfluidic fabrication is molding, which is a method based on reproducing the microchannel design using a master mold (Figure 4B). It can be divided into four categories: replica molding, injection molding, hot embossing, and wire molding. The replica molding method, Figure 4B­(i) (e.g., soft lithography when using soft polymer-based master molds), is the most usual fabrication technique for OoCs and generally follows the steps: (i) create a pattern using a computer-aided design and develop a master mold; (ii) add PDMS in the mold; (iii) cure it; (iv) remove the PDMS and bond to a glass slide.^110^ This method was first used to create a lung-on-a-chip in 2010, and since then, it has been widely applied to other tissues.^111,112^ It can reach microchannels ranging from 20 μm in size to 100 μm in size.

In BBB-OoC models, soft lithography has been reported recently to produce microdevices inspired by the lung-on-a-chip configuration, with channels separated by porous membranes,^113–116^ and to produce a design with parallel channels separated by micropillars.^33,117–119^ Based on this, Ahn and collaborators used soft lithography to produce a microengineered human BBB platform to understand the transport mechanism of high-density lipoprotein nanoparticles. They demonstrated the distinct cellular uptakes and BBB penetrations through receptor-mediated transcytosis.^113^ Shi et al. explored this technique to develop a BBB-glioma microfluidic chip model to evaluate the permeability and antiglioma activity. They examined the potential of using a combination of traditional Chinese medicine compounds (i.e., matrine, wogonin, paeoniflorin, osthole, resveratrol, and quercetin) as a multitarget and multicomponent approach for cancer therapy. Their findings showed that the BBB hindered the absorption of drugs and their ability to kill glioma cells.^33^

Due to the limited scalability of PDMS-based chips and the high material costs, manufacturing technologies using thermoplastics such as PMMA, COC, polystyrene (PS), polyether ether ketone (PEEK), polyethylene terephthalate (PET), and polypropylene (PP) have been used as alternatives to soft lithography in OoC fabrication.^120,121^ Injection molding, Figure 4B­(ii), is a standard method for processing polymers in different molds. The process involves four main steps: (i) melting the thermoplastic until it becomes a liquid state; (ii) compressing the two halves of the mold to create a mold cavity; (iii) injecting the thermoplastic to fill the mold cavity; and (iv) cooling the mold, removing the cast part, and thermal bonding it.^100,107^ To improve the scalability of PDMS-based microfluidic devices, Lee et al. used injection molding to construct a PS molded array 3D neuron culture platform with a standard 96-well plate form factor to recapitulate central and peripheral nervous system elements. They cocultured neurons, hBMECs, and ACs to validate the model as a high-throughput screening platform to engineer the neuronal microenvironment.^122^

Hot embossing, Figure 4B­(ii), is similar to injection molding, as it involves melting thermoplastics and shaping them into molds using heat and pressure. This process consists of three stages: (i) the polymer is inserted between the molds and heated above the glass transition temperature; (ii) the molds are pressed against the polymer; and (iii) all parts are cooled; the processed polymer is demolded and thermal bonded.^103,123^ Despite advantages such as costs and simplicity, the limitations of both methods include the restriction of thermoplastic materials and their disadvantages (i.e., low transparency for images) and the requirement for a master mold fabrication previously^107^ Another issue surrounding the use of PDMS in a biological context is the absorption of small and hydrophobic molecules, which can limit some applications (i.e., applications involving hormones).^124^ In this sense, Jeon et al. developed a microfluidic device with enhanced 3D gel capabilities made with COC using hot embossing from an epoxy master. They used collagen I as a scaffold for hMVECs, obtaining no adverse effects in cell viability as compared to previous PDMS devices.^125^

Wire molding, Figure 4B­(iii), is an effortless and common technique for creating circular microchannels. The wire molding generates circular cross sections by casting PDMS or other materials around different templates, such as needles, nylon threads, glass rods, or metal microwires.^101,105^ Microchannels can be created by embedding microwires in the material and removing the wires after curing. This technique offers easy fabrication and access to small sizes.^101,126^ This method is often reported as BBB mimicking due to the circular cross-section design, as observed in vivo.^127^ In these cases, hydrogels embed the wire template, on which the cells are cultured. As the brain has a soft tissue structure, natural hydrogels from ECM (i.e., collagen I, hyaluronic acid, and fibrin) are commonly used to mimic this complex microenvironment. Recently, collagen type I was reported by some groups as the material used to mold the microchannels due to its similarity with the ECM. Using microneedles, Seo and collaborators developed a microfluidic device using collagen I to coculture hBMECs with human ACs and human brain vascular PRCs.^34^ Also, collagen I was micromolded combined with Matrigel and other extracellular protein matrices (such as fibronectin and collagen IV) to mimic brain microvessels, using nitinol wire as a sacrificial mold.^128–130^

### 3.3 3D Printing

3D printing is a manufacturing process that adds material layer by layer to create a 3D object. Fabricating microfluidic devices using 3D printing can be highly efficient since it can be used to fabricate robust devices, print master molds to construct microchips by casting PDMS, or distribute living cells in a definite pattern (i.e., bioprinting), as shown in Figure 4C.^100,131–133^ Fabricating PDMS-based devices using 3D-printed master molds has several advantages over traditional fabrication methods such as soft lithography and keeps the attractive properties of PDMS, such as oxygen permeability, while not requiring a clean room.^134^ This section focuses on three 3D printing technologies: fused deposition modeling (FDM), stereolithography (SLA), and 3D bioprinting.

FDM 3D printers, Figure 4C­(i), heat and melt a thermoplastic filament and then extrude it through a nozzle to create a 3D object. The liquid material is deposited on the build platform and then cools and solidifies, repeating in a layer-by-layer process. It is probably the most recognized 3D printing method and works with inexpensive biocompatible polymers.^132,134^ SLA, Figure 4C­(ii), is an additive manufacturing process that works through an optical process of building layer upon layer. It is possible to quickly produce high-quality features by using a polymerized resin vat and a structured light source (in which UV light is prevalent) to create each layer.^131,134^ The method should be chosen based on the expected resolution: FDM printers usually reach 125 × 125 × 200 μm (*X* × *Y* × *Z*) resolution, and SLA printers can get 56 × 56 × 50 μm, both depending on the printer mold and the material used.^135^

Recently, 3D-printed molds have been reported to construct BBB-on-a-chips. For instance, Lyu and collaborators used a master mold fabricated using SLA to build a neurovascular unit to study stem cell therapies in ischemic stroke.^136^ Hajal et al. used a 3D printed template for the macro devices to construct an engineered BBB microfluidic model for vascular permeability analysis, in which the technology used for printing was not specified.^137^ 3D-printer mold was also reported by Wang et al. to develop a microfluidic system to investigate the combinatory effect of photothermal treatment and photo-oxygenation in the inhibition of Aβ-aggregation in Alzheimer’s disease.^138^

3D bioprinting, Figure 4C­(iii), is a promising strategy that uses biomaterial-encapsulated living cells to create complex 3D structures with high accuracy and precision, in which cell-laden biomaterials are called bioinks.^139^ PDMS has historically been the most common OoC structural material. Still, hydrogels have been introduced into the OoC field to achieve specific mechanical properties and biochemical stimuli response.^140^ In recent years, bioprinting and microfluidics have been combined to construct 3D models such as tissues and organs.^141^ Therefore, biocompatible hydrogels, such as alginate, gelatin, collagen, gellan gum, fibrin, and gelatin methacryloyl (gelMA), are usually used as bioinks to encapsulate cells, protecting them from the shear forces that occur during the printing process.^133,142^

Researchers have utilized microfluidic channels and chambers as the receiving plate from printed bioink to carry out 3D printing on a chip.^139^ This method enables printing, culture, administration of stimuli, and detection of 3D constructs. Advanced techniques for 3D printing on a chip can generate hydrogel-based flow networks that closely resemble natural vessels in form and function.^143^ Even if 3D printing on a chip was not reported recently in BBB research, other applications were observed. For instance, Zhang et al. developed a PDMS chip with 3D-printed alginate hydrogel for multiple cell patterning for drug metabolism and diffusion studies.^144^ Hamid and the group used an SLA 3D-printer chip to receive gelMA hydrogel for long-term cell culture to investigate cancer cells in a cocultured microfluidic environment.^145^ Abudupataer and collaborators developed a vessel-on-a-chip using a laser cutter to construct a PMMA chip, in which primary human aortic endothelial cells (HAECs) and human aortic smooth muscle cells (HASMC) were printed with gelMA as bioink.^146^

Printing individual microfluidic channels and channel networks inside the hydrogel compartment is possible by utilizing sacrificial (or fugitive) material. The four steps involved in sacrificial material printing are (i) 3D printing the sacrificial material, (ii) casting or printing hydrogel (cell-laden) around the sacrificial material, (iii) cross-linking the hydrogel to make it stable, and (iv) removing the sacrificial material.^140,147^ In this sense, Yue et al. described a 3D vascularized neural construct for the reconstitution of BBB function. They developed a microfluidic system, which was made using a 3D bioprinting sacrificial template (solution with alginate, gelatin, and Pluronic F-127). After the template solidified, it was coated with a PCL/PGLA solution. The sacrificial template was removed, and the PCL/PLGA was coated with collagen to improve cell adhesion.^148^

Despite the many fabrication methods available (such as molding, laminate manufacturing, and 3D printing), molding-based techniques remain the most commonly used for the fabrication of the OoC and BBB-OoC, emphasizing soft lithography. Combining these methods has enabled the creation of more complex in vitro models (for example, using molding methods with 3D printed molds), focusing on reducing the cost of OoC fabrication methods and incorporating new materials. As a result, engineering aspects, such as microchip designs, different hydrogels, and materials, have been researched in the BBB-OoC field and will be further explored in the following section.

## 4 BBB-on-a-Chip: Characterization, Engineering Aspects, and Designs

Recent advances in microfluidic and biological research have allowed us to monitor and reproduce aspects of the BBB (such as dynamic and complex interactions between the cells) on a chip platform, overcoming the gap between in vitro BBB models. For example, BBB-on-a-chip models have shown the importance of shear stress on the ECs for their differentiation and formation of tight junctions, as well as the cell-to-cell interactions for accurately inducing in vivo physiological responses.^149,150^ To mimic the BBB, the microfluidic platforms have enabled methods to assess BBB integrity and barrier function, such as measurement of TEER, permeability assays, or immunostaining.^151^

### 4.1 Monitoring and Characterizing the BBB

#### 4.1.1 Transendothelial Electrical Resistance (TEER)

The tight junctions formed between ECs in the brain microvasculature restrict the movement of molecules, including small ions such as Na^+^ and Cl^–^, creating a measurable electrical resistance called TEER. To obtain the TEER value, two types of measurements can be applied: (i) ohmic resistance analysis and (ii) impedance spectroscopy (Figure 5). In the first case, basically, electrodes are placed on opposite sides of the BBB—one on the blood side and the other on the brain side, in which the obtained total electrical resistance includes the resistance of the cell layer (*R*_TEER_), the cell culture medium (*R*_M_), and the semipermeable membrane insert (*R*_MEMB_).^152,153^ TEER values represent the paracellular permeability of the monolayer and, in the simplest case, for ohmic resistance, are calculated as eq 1

1 $$TEER=R_{TEER}\times A\;\left[\Omega \;\cdot {cm}^{2}\right]$$

*R*_TEER_ represents the resistance of the endothelial barrier, while *A* is the common area of the top and bottom channels between the reference and working electrodes.

**Figure 5:**
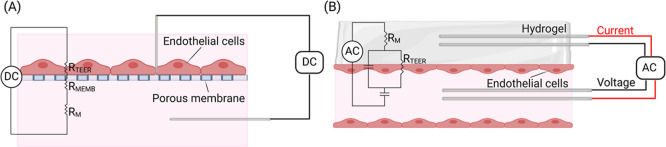
TEER measurements by (A) ohmic resistance and (B) impedance spectroscopy. DC: direct current; *R*_TEER_: resistance of endothelial barrier; *R*_MEMB_: resistance of porous membrane; *R*_M_: resistance of culture medium; and AC: alternating current (created using Biorender.com).

Real-time TEER measurements can be performed without cell damage using direct current (DC) voltage to measure ohmic resistance and the resulting current (eq 1, Figure 5A).^152,153^ However, designs without porous membranes and hydrogel-based models (which will be exposed in the next section) cannot deliver the TEER value-based evaluation of the EC layer through ohmic resistance, because they cannot provide TEER measurements. Introducing the electrode into the “blood” channel of the hydrogel device can easily disrupt the EC layer.^154,155^

Impedance spectroscopy (Figure 5B) is a noninvasive technique that provides more electrical parameters in the biological barrier (i.e., the capacitance of the cell layer), making it possible to fully characterize the studied cell system.^156,157^ It involves applying a small amplitude alternating current (AC) excitation signal with a frequency sweep and measuring the amplitude and phase responses of the resulting current.^158,159^ The electrical impedance (*Z*) is a time-function ratio between the voltage perturbation (*V*(*t*)) and the resulting current (*I*(*t*)) (eq 2) and can be used to quantify the TEER. Mori et al. (2018) fabricated a system to measure the TEER of the hydrogel-based channel by embedding a syringe needle adhered with a pair of electrodes in the 3D hydrogel, extracting the needle to form a hollow channel, and introducing the electrodes simultaneously. With the electrode pairs placed inside the 3D channel, the TEER of an endothelial layer formed on the 3D channel could be measured (eq 2, Figure 5B).^154^

2 $$Z=\frac{V\left(t\right)}{I\left(t\right)}\left[\Omega \right]$$

#### 4.1.2 Permeability Assay

An alternative method for testing the tightness of the BBB is the molecule permeability test, which measures the diffusivity of a fluorescent probe across the barrier through spectroscopy (for planar chips) or image analysis (for hydrogel-based chips). A lower permeability coefficient indicates superior barrier properties. The permeability associated with the passive diffusion of a solute across a cell monolayer can be obtained from Fick’s law.^160^ The permeability may be calculated according to the microfluidic designs: planar or cylindrical geometries. The geometry is better described in the following sections. The permeability across planar configurations can be computed as

3 $$P=\frac{1}{C_{l}}\times \left[\frac{d\left(C_{b}\times V_{b}\right)}{dt}\right]\times \frac{1}{A}$$

which can be simplified for short times (*C*_b_ ≪ *C*_l_) as

4 $$P=\frac{C_{b}}{C_{i}}\times \frac{V_{b}}{A}\times \frac{1}{t}$$

where *C*_b_ is the concentration of tracer in the brain, *C*_l_ is the concentration in the vascular channel, *V*_b_ is the volume in the brain channel, *A* is the contact area between two compartments, and *t* is the perfusion time.^15,160,161^ Conversely, in cylindrical configurations, the permeability is usually measured from fluorescence images. In the simplest case, the fluorescence intensity is linearly proportional to concentration, and the permeability can be calculated as

5 $$P=\frac{1}{\Delta I}\times \left(\frac{dI}{dt}\right)\times \frac{d}{4}$$

where Δ*I* represents the initial step increase in fluorescence intensity, d*I*/d*t* is the initial rate of increase in fluorescence intensity as the solute begins to diffuse, and *d* is the diameter of the vessel.^162^

Alternatively, the permeability can be represented through the penetration ratio, obtained by dividing the concentration of the tracer penetrating into the brain channel by the initial total concentration (added in the blood channel) (eq 6)^163^

6 $$penetration\;\left(\%\right)=\frac{C_{b}}{C_{T}}\times 100$$

One of the most common tracer molecules used for permeability assays is the fluorescein isothiocyanate (FITC)-labeled dextran, which may vary from 3 to 2000 kDa in molecular weight.^161,164^ Permeability assessment is significantly affected by the molecular weight of dextran, which is transported passively through the paracellular pathway via hydrophilic molecular complexes that pass through the spaces between adjacent EC across the BBB.^164,165^ Other trace molecules used in permeability studies include FITC-labeled sucrose (353 Da) and mannitol (187 Da).^46^ Additionally, researchers also use small molecule dyes, such as sodium fluorescein (376 Da), Lucifer yellow (444 Da), and Cascade Blue (530 Da), as probes for BBB integrity measuring.^128,137^ Also, permeability experiments were used to determine the paracellular transport of antibodies and drugs.^166^

#### 4.1.3 Immunostaining

Immunofluorescence can be used to measure the expression of specific tight junction (TJ) and adherens junction (AJ) proteins by ECs (Figure 6A,B, respectively). These proteins include occludins, claudins, zona occludens (ZO) proteins, junctional adhesion molecules (JAMs), cadherins, and nectins.^167,168^ As the first integral membrane protein discovered in TJ (Figure 6A), occludin is the most expressed and reliable immunohistochemical marker.^165,169^ Claudins (Figure 6C­(iii)) establish homophilic and heterophilic interactions via extracellular loops, forming the backbone of TJs.^170^ In addition, ZO-1 and ZO-2 are critical for junction assembly and the clustering of claudins and occludin, leading to the formation of tight junctional strands.^171,172^ JAMs are crucial transmembrane components of TJs, controlling the passage of nutrients and solutes across an EC monolayer and modulating many cellular functions, including cell polarity, cell migration, proliferation, and paracellular permeability.^173^

**Figure 6:**
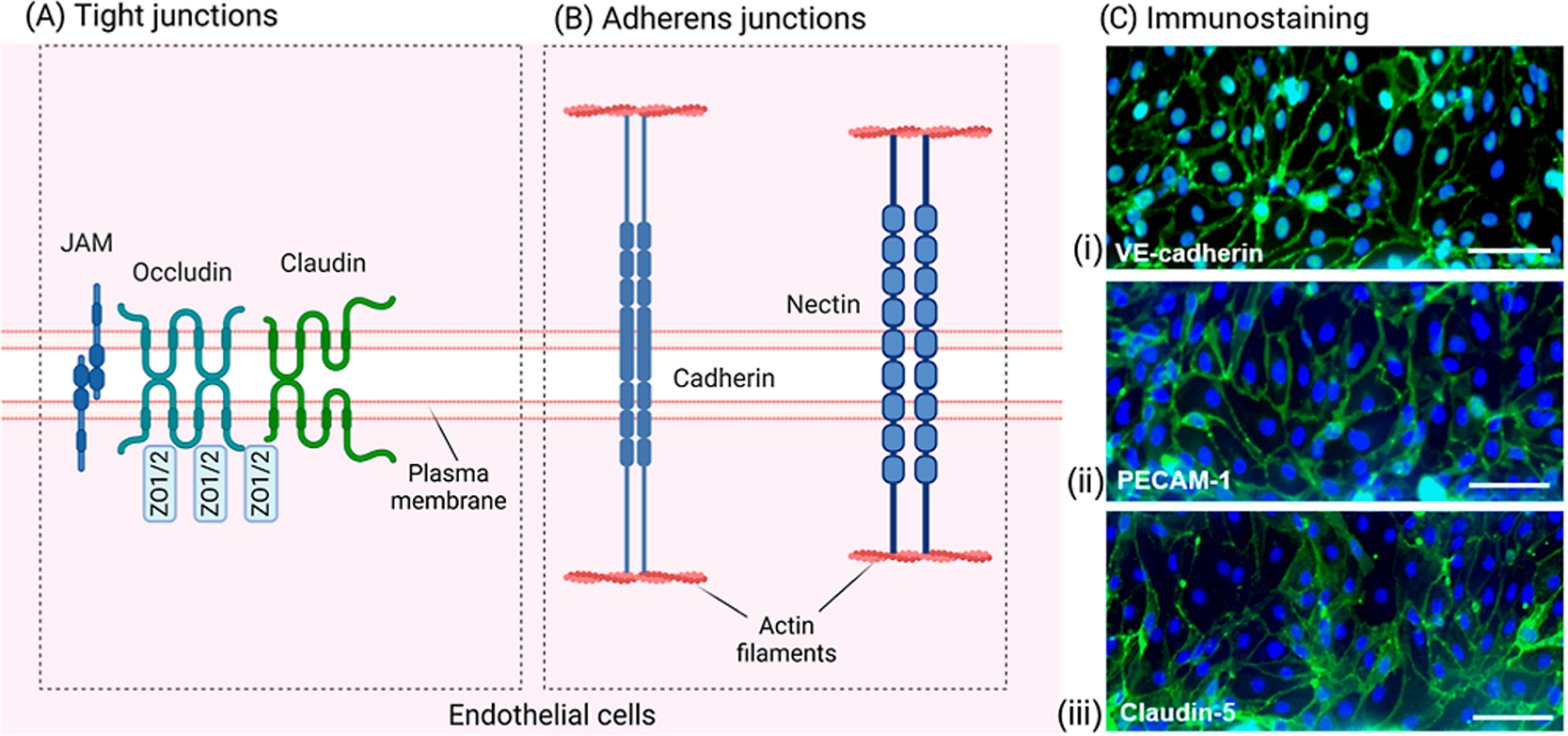
Endothelial cells (A) tight junctions (connecting areas of plasma membrane), (B) adherens junctions (joining the actin filaments) (created using Biorender.com), and (C) immunostaining of human brain microvascular endothelial cells for nuclei (blue) and for adherens and tight junction proteins (green) in BBB-on-a-chip: (i) vascular endothelial cadherin (VE-cadherin), (ii) platelet endothelial cell adhesion molecule 1 (PECAM-1), and (iii) claudin-5 at the cell–cell contacts (scale bar = 50 μm). Adapted from.^174^ Available under a CC-BY 4.0 license. Copyright 2018, Nienke R. Wevers et al.

Cadherins (Figure 6C­(i)) are important adhesive molecules in AJs (Figure 6B), connecting cells and linking intercellular adhesion structures with the cytoskeleton.^175^ Nectins provide the initial framework for the formation of AJs and TJs.^176^ In addition to TJs and AJs proteins, the expression of the P-glycoprotein is also assessed, which regulates the absorption of hydrophobic molecules as an efflux pump. The evaluation of EC markers has been crucial in researching the BBB, and combining it with the microfluidic system showed significant advantages, including factors like shear stress and appropriate cell–cell interactions.^177,178^

### 4.2 Microfluidic Designs, Engineering Aspects, and Applications

Considering the engineering aspects, some microfluidic designs have been reported in the literature and can be generally divided into planar and cylindrical designs. Here, we categorized the planar design into vertical configuration (also named vertical design or sandwich design), with channels separated by a porous membrane (Figure 7A,B), and horizontal configuration, with channels separated by micropillars or hydrogel barriers (Figure 7C). The following section will explore and summarize the main designs used to mimic the BBB, along with their applications over the past four years. Engineering aspects such as chip configuration, porous membrane material, coating, and hydrogel choice will be discussed, as well as the recent biological applications and obtained results for these models.

**Figure 7:**
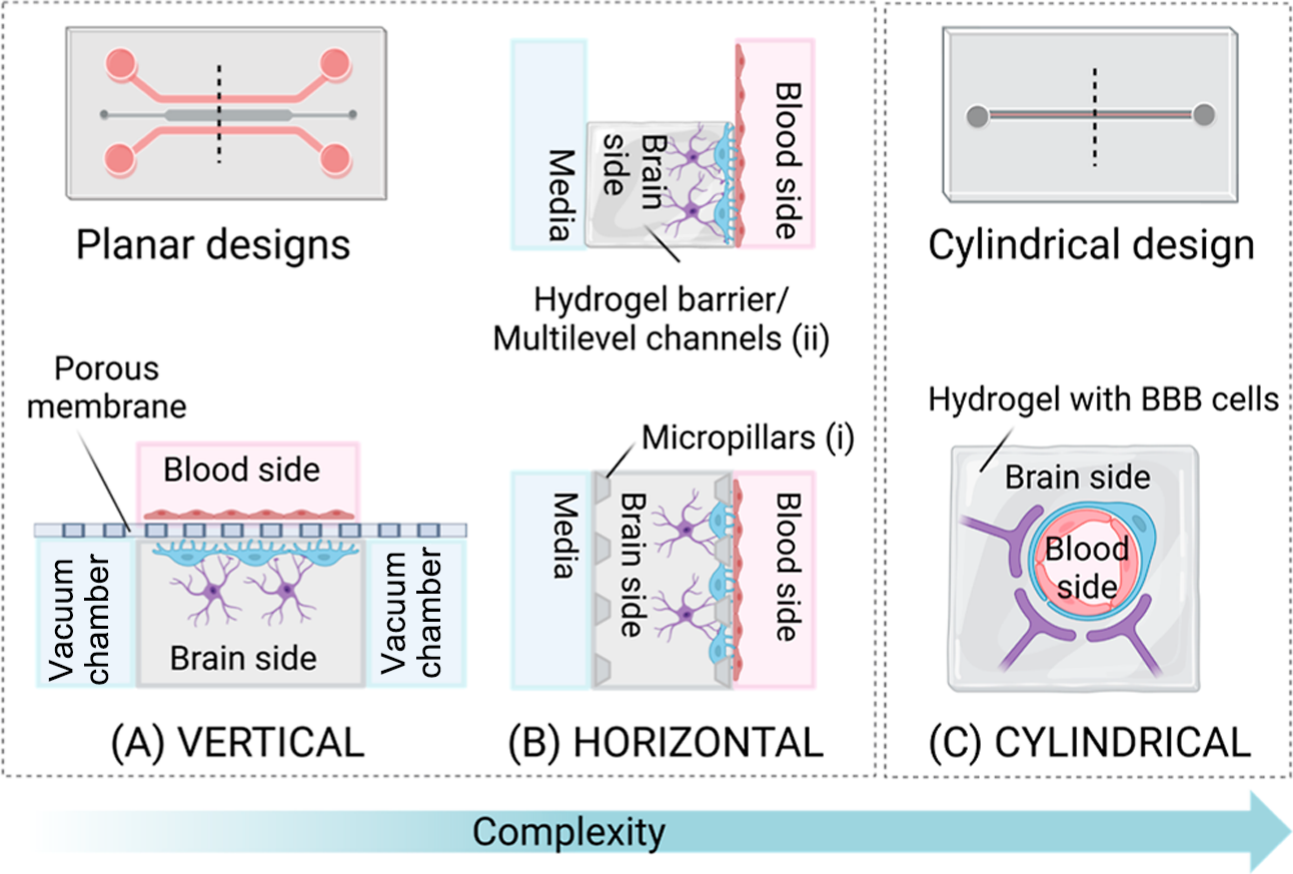
Microfluidic chip designs: planar design with (A) vertical configuration and (B) horizontal configuration with (i) micropillars and (ii) hydrogel barrier and (C) cylindrical design (created using Biorender.com).

#### 4.2.1 Planar Design with a Vertical Configuration

BBB models with a vertical design share many similarities with the traditional transwell assay. They are composed of the blood side (apical) with brain endothelial cells (BECs) and the brain side (basolateral) with another BBB cell type, such as PRCs, ACs, or neurons.^179^ These microfluidic chips are derived from the lung on a chip reported in 2010, which consists of a porous membrane placed between PDMS chambers that are bonded to a glass side, generally constructed by soft lithography or xurography.^111,180^ The porous membrane acts as a barrier between the apical (ECs) and basolateral sides (brain cells), similar to the basement membrane in living organisms. Its semipermeable nature enables biochemical and physical exchange between cells and is a suitable platform for coculture.^181,182^ Generally, BECs are cultured on the upper side of the porous membrane, and flow is typically connected to this chamber to apply shear stress to these cells.^183^

Choosing the material for porous membranes is challenging because it involves homeostasis, cell structure support, cell differentiation, and tissue maintenance. It is ideally made of biocompatible materials with a small thickness.^165^ In recent years, synthetic membranes, such as polycarbonate (PC), polyethylene terephthalate (PET), and PDMS membranes, have often been used for BBB models. However, the hydrophobic nature of these materials leads to poor wetting and cell adhesion.^184^ To address this issue, surfaces are typically coated with ECM proteins (i.e., collagen, laminin, and fibronectin), commercial basement membrane components (like Matrigel and Geltrex), or poly­(amino acids) (i.e., poly-l-lysine and poly-d-lysine) to provide a more natural environment for cell attachment and survival.^184–186^ Table 3 describes all the sandwich-design microchips reported in the last four years for BBB models, highlighting their membrane material and coating, fabrication method, cell type, and BBB characterization.

**Table 3:** Recent Publications of BBB-on-a-Chip with a Planar Design and Vertical Configuration

	material			BBB cells		BBB characterization				
fabrication methods	chip	membrane	coating	apical	basolateral	TEER (Ω cm^2^)	permeability (×10^–6^) (cm s^–1^)	diffusion coefficient	penetration ratio (%)	ref
commercial chip (Emulate, Inc.)	PDMS	PDMS	collagen IV, laminin, and fibronectin	hiPSC-derived glutamatergic and GABAergic neurons, hACs, iPSC-derived microglia, and hPRCs	iBMECs	N/A	FITC-dextran (3 kDa):	N/A	N/A	^48^
							•with iBMECs: 1.5			
							•without iBMECs: 6			
molding (soft lithography)	PDMS	PET	collagen IV and fibronectin	mACs and mPRCs	mBECs	control (transwell)	FITC-dextran (10 kDa):	N/A	N/A	^201^
						day 1: 100	•transwell: 0.18			
						day 9: 300	•chip: 0.06 (ratio of permeability)			
commercial chip (Emulate, Inc.)	PDMS	PDMS	collagen IV, laminin, and fibronectin	hBVPs and hACs	hBMECs	N/A	caffeine: 29.98	N/A	N/A	^202^
							carbamazepine: 15.77			
							cetirizine: 7.29			
							desipramine: 32.45			
							donepezil: 15.75			
							loperamide: 9.25			
							nefazodone: 3.84			
							simvastatin: 6.12			
3D printing and bioprinting (hydrogel)	PMMA and stainless steel	PET	collagen type I	hCMECs, hBVPs, ACs, NPCs and hMC3	hBVPs and ACs	day 3: 250	N/A	FITC-dextran (10 kDa):	N/A	^193^
						day 5: 280		day 3: 0.22		
						day 7: 300		day 5: 0.15		
								day 7: 0.18		
molding (soft lithography) and 3D printing	PDMS	PET	Matrigel	hCMECs	N/A	N/A	N/A	N/A	l-Dopa with different shear stress:	^163^
									–static BBB:	
									•0.6 dyn cm^–2^: 3	
									•3.0 dyn cm^–2^: 3.5	
									•13.0 dyn cm^–2^: 5	
									–dynamic BBB (0.10 μL min^–1^):	
									•0.6 dyn cm^–2^: 2	
									•3.0 dyn cm^–2^: 2.5	
									•13.0 dyn cm^–2^: 4	
molding (soft lithography)	PDMS	PET	collagen type I	hCMECs	hACs and hMC3	N/A	FITC-dextran (10 kDa): 1.6	N/A	N/A	^114^
molding (3D-printed mold)	PDMS	PET	collagen IV and fibronectin	HUVECs	hACs	N/A	FITC-dextran:	N/A	N/A	^138^
							•3 kDa: 1.8			
							•10 kDa: 0.225			
							•70 kDa: 0.341			
molding (soft lithography)	PDMS	PET	collagen type IV and collagen type I	iBMECs	hACs	N/A	N/A	N/A	N/A	^203^
molding (soft lithography)	PDMS	PC	Matrigel	ECs	N/A	N/A	N/A	N/A	N/A	^190^
commercial chip (Emulate, Inc.)	PDMS	PDMS	collagen type IV and fibronectin	hACs and PRCs	iBMECs	N/A	–static BBB:	N/A	N/A	^46^
							•sucrose: 1.521			
							•mannitol: 1.105			
							–dynamic BBB (3 dyn cm^–2^):			
							•sucrose: 0.84			
							•mannitol: 0.8713			
molding (soft lithography)	PDMS	PDMS	laminin	iBMECs	N/A	N/A	N/A	N/A	N/A	^115^
commercial chip (Emulate, Inc.)	PDMS	PDMS	collagen IV, fibronectin, and laminin	iBMECs	iPSC-DA neurons; hACs, PRCs, and microglia	N/A	FITC-dextran (3 kDa): range of 1–3;	N/A	N/A	^198^
							Lucifer yellow: range of 4–6			
molding (soft lithography)	PDMS	PC	fibronectin, poly-l-lysine, and Matrigel	hBMECs	hBVPs and hACs	static BBB: 110	FITC-dextran (4 kDa and 40 kDa): 1	N/A	N/A	^113^
						dynamic BB:				
						•0.4 dyn cm^–2^: 120				
						•4 dyn cm^–2^: 150				

PC membranes with different pore sizes (ranging from 400 nm to 8 μm) are commonly used as artificial basement membranes in microfluidic devices due to their similarity to those found in commercial transwells, enabling shear stress studies into robust cell adhesion.^187,188^ However, this material presents some drawbacks. Its poor optical transparency makes monitoring biomolecular transport and cell attachment in real time difficult, requiring fluorescence staining and preventing observation over time.^165,181,188^ Also, PC membranes require additional chemical treatments to bond with the PDMS chamber, e.g., surface activation with oxygen plasma or functionalization with silane molecules (such as (3-aminopropyl) triethoxysilane (APTES), (3-glycidyloxypropyl) trimethoxysilane (GPTMS), or (3-aminopropyl) trimethoxysilane (APTMS)).^182,189^ Ahn et al. (2020) utilized soft lithography to create a microengineered human BBB platform, as illustrated in Figure 8A­(i). This platform consisted of a PC coated with fibronectin, poly-l-lysine, and Matrigel arranged in a vertical design with micropillars. The platform was used to study the mechanisms of nanoparticle transport by employing high-density lipoprotein (HDL)-mimetic nanoparticles labeled with fluorescence (Figure 8A­(ii). They obtained the BBB structure and function using ECs, ACs, and PRCs, demonstrating the cellular uptakes and BBB penetrations (by adding the NPs on the upper side, measuring the fluorescence intensity on the lower side) and capturing 3D nanoparticle distributions at cellular levels using confocal microscopy (Figure 8A­(iii).^113^ Jeong et al. (2021) also used soft lithography to develop a microchip with a PC membrane to investigate the formation of tight junctions associated with shear stress. They also developed a numerical approach using computational fluid dynamics to predict the in vivo level shear stress for the microfluidic BBB-on-a-chip, varying conditions such as the flow rate, the porosity of the PC membrane, and the dimensions of the microfluidic channel. The numerical simulation approach predicted shear stress with a 2.17% error rate compared to the experimental results.^190^

**Figure 8:**
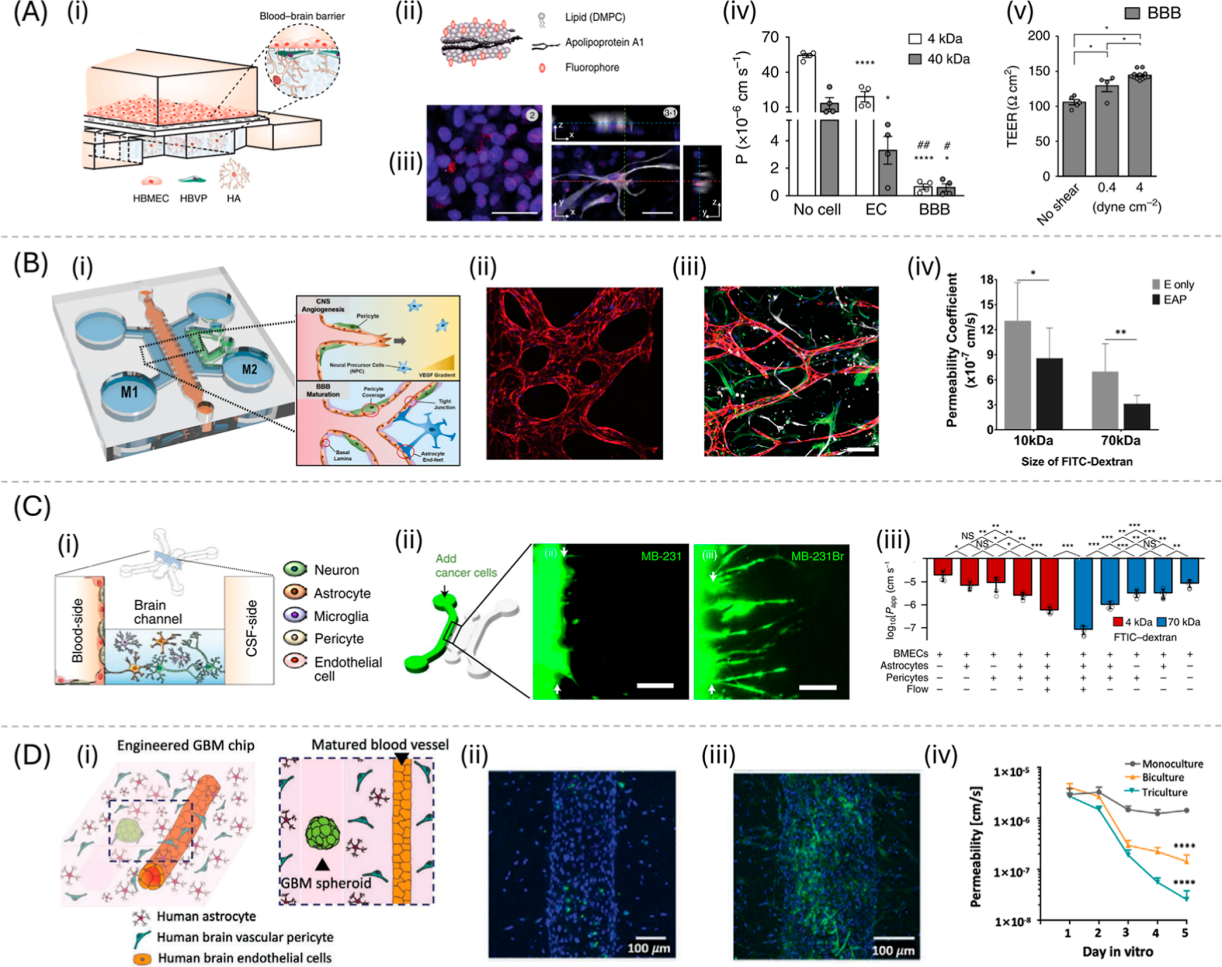
BBB-on-a-chip designs: (A) planar design with a vertical configuration and PC membrane. (i) Schematic illustration of vertical configuration; (ii) engineered HDL-mimetic nanoparticle consisting of lipid, apolipoprotein A1 (eHNP-A1), and fluorescent marker; (iii) confocal images showing eHNP-A1 within the HBMEC monolayer and HAs in a BBB chip; (iv) permeability coefficients (cm s^–1^) calculated from the diffusion of 4 and 40 kDa FITC-dextran; and (v) TEER measured from BBB models under different levels of shear stress. Reproduced from.^113^ Available under a CC-BY 4.0 license. Copyright 2020, Ahn et al. (B) Planar design with a horizontal configuration and micropillars. (i) Schematic illustration of horizontal configuration with micropillars and the perivascular cells; Confocal images of 3D vasculature with (ii) monoculture with HBMECs and (iii) triculture with HBMECs, PRCs, and ACs (scale bar = 200 μm); and (iv) permeability coefficients (cm s^–1^) of each culture condition (E = HBMEC only and EAP: HBMECs, PRCs, and ACs) calculated from the diffusion of 10 and 70 kDa FITC-dextran. Reproduced with permission from.^194^ Copyright 2019 John Wiley and Sons 2019, Lee, Chung, Lee et al. (C) Planar design with a horizontal configuration and multilevel. (i) Schematic illustration of the horizontal configuration with multilevel and the BBB cells; (ii) incorporation of cancer cells (MB-231 and MB-231Br) and the extravasation across the BBB. The white arrows indicate the hydrogel boundary (scale bars = 100 μm); and (iii) log-transformed value of the apparent permeability coefficients, *P*_app_, of the endothelium in the chips. Reproduced with permission from.^136^ Copyright Springer Nature 2021, Lyu et al. (D) Cylindrical design. (i) Schematic illustration of engineered glioblastoma multiforme (GBM) chip; confocal images showing the expression of ICAM1 (intercellular adhesion molecules, green) in (ii) BBB chips and (iii) tumor chips; and (iv) permeability coefficient (cm s^–1^) over a period in a culture of different culture conditions. Reproduced from.^34^ Available under a CC-BY 4.0 license. Copyright 2021, Seo, Nah, Lee et al. Advanced Functional Materials published by Wiley-VCH GmbH.

PET membranes have been used as an excellent replacement for PC membranes to optimize the transparency of the porous membrane and consequently improve the optical transparency, which is essential to acquire images during biological assays, maintaining the same range of pore size (400 nm to 8 μm).^181,191^ However, both PC and PET membranes have poor adhesion between PDMS layers, requiring additional treatments.^192^ Vertical configuration with a PET membrane coated with collagen type I was reported by Wang et al. (2022) in a platform for studies of brain metastasis of tumors in vitro, using soft lithography as the fabrication method. They established a human BBB model by coculturing ECs, ACs, and microglial cells to explore the potential role of exosomes derived from malignant melanoma in modulating BBB integrity.^114^ Recently, Xu and collaborators reported using PET membranes to construct a parallel multilayered platform for screening chemotherapeutical drugs (vorinostat and 5-fluorouracil) using a 3D-printed microchip associated with 3D bioprinting. The microfluidic device is composed of two parallel membranes separated by a hydrogel layer (composed of gelMA, gelatin, fibrinogen, and laminin). The first membrane was placed in a stainless-steel sheet after cell seeding (ECs, PRCs, ACs, microglia, and neural progenitor cells), and the hydrogel was added through bioprinting, followed by the second membrane, the stainless-steel sheet, and the PMMA layer. The researchers demonstrated that the hydrogel they used has mechanical properties similar to those of brain tissue, with an elastic modulus ranging from 100 to 3000 Pa. They also used FITC-dextran (10 kDa, 40 kDa, and 70 kDa) to illustrate that the microchip offers a dependable platform for conducting thorough studies on permeability.^193^

As an alternative to PC and PET stiff membranes, porous flexible membranes can be easily fabricated with different pore sizes using PDMS (generally with 7 μm of pore size and 50 μm of thickness) according to protocol development by.^195^ It has an easy manufacturing process and has advantages, such as higher biocompatibility and transparency than other materials. Also, it eliminates the need for additional chemical treatment to bond the membrane with the PMDS chip.^12,196^ In addition, due to PDMS’s polymer network structure, this membrane also has high permeability, enabling oxygen supply and carbon dioxide removal.^12,197^ Noorani et al. (2021) used a commercially available chip from (Model N/A, Emulate, USA) composed of a PDMS porous membrane for brain permeability studies. To increase the cells’ adhesion, the surface of the PDMS was coated with collagen IV and fibronectin, and ACs and PRCs were seeded in the apical channel and iBMECs (iPSC-derived BMECs) in the basal channel, obtaining a highly predictive and translationally relevant BBB model.^46^ To understand Parkinson’s disease, Pediaditakis et al. (2021) also used a commercial chip with a PDMS membrane (Model Chip-S1, Emulate, USA) to recreate the human brain-on-a-chip. Their alpha-synuclein aggregates model containing dopaminergic neurons, ACs, microglia, PRCs, and ECs could reproduce several critical aspects of Parkinson’s disease, such as mitochondrial impairment, neuroinflammation, and compromised barrier function.^198^ A PDMS membrane device was also used to study the reduction of triple-negative breast cancer (which has a high propensity for metastasis to the brain) through systemic ligand-mimicking bioparticles (NNCs) cross the BBB.^115^ The microfluidic chip was fabricated by soft-lithography and validated with the NNCs carrying tumoricidal agents (e.g., oligonucleotide duplexes with doxorubicin) to reduce the growth of intracranial tumors and to improve the therapeutic profile compared to current therapeutic interventions (e.g., liposomal doxorubicin formulation).

Regarding the BBB characterization, the same range of permeability values, 1 × 10^–6^ cm s^–1^, was observed in all vertical configurations (sandwich design), Table 3, independent of membrane materials PC, PET, and PDMS and FITC-dextran molecular weight (from 3 to 40 kDa). Compared with ECs cultured alone, a triculture with PRCs and ACs significantly decreased the BBB leakage of FITC-Dextran, as reported by Ahn (2020), illustrated in Figure 8A­(iv) and Noorani (2021). Yuan et al. quantified the in vivo permeability in rat cerebral microvessels, observing a range of 1.5–9.2 × 10^–7^ cm s^–1^ to different FITC-dextran molecular weights (4–70 kDa), showing that the permeability coefficient reached in the microfluidic models were slightly higher than as the measured in vivo.^199^ Due to the challenge of associating the TEER measurement with the BBB chips, only a few studies have reported this analysis. Xu et al. reported the correlation between the obtained TEER values and the period of time, showing that there is an increase during 7 days.^193^ Another study investigated the effect of the flow rates on TEER values (Figure 8A­(v)), they obtained data varied from 100 to 300 Ω cm^2^.^113^ However, the obtained values were lower than those observed in vivo, with expected values between 1500–8000 Ω cm^2^,^200^ showing that there is still a discrepancy between these models and what is observed in vivo.

Recent publications have discussed planar designs with vertical configurations, as shown in Table 3. A notable trend is the variety of methods used in the field. While soft lithography using PDMS remains the most commonly used microfabrication method, recent works have shown the integration of other methods, such as bioprinting. Commercial chips, such as those sold by Emulate, have also been reported, indicating the diversity of approaches in our field.

#### 4.2.2 Planar Design with a Horizontal Configuration

Brain-mimicking microfluidic chips with planar design in horizontal configuration overcome the drawbacks of vertical design, enabling better observation and easier fabrication.^204,205^ Even in the vertical configuration, the horizontal design is rectangular, resulting in flow motions and wall shear stress that contrast in vivo brain capillaries.^127^ This design replaces the porous membrane between blood and brain channels with PDMS channels separated by micropillars (called micro posts too) or hydrogel barriers, as shown in Figure 7B.^165,206,207^

The micropillars usually have a trapeze form and are constructed by soft lithography with different dimensions. Often, it is used in combination with ECM hydrogels to increase cell viability.^208,209^ On the other hand, the channels in the horizontal layout can be separated only by hydrogel without any other physical structure, a design also known as multilevel channels. For that, the channels have different levels, forming a step in which the hydrogel is added to act as a semipermeable barrier, and fabrication could be made with some methods such as soft lithography, 3D printing, or xurography.^210^ Compared to micropillar barriers, the multilevel channels may reduce some defects that occur in forming the continuous and intact endothelium, affecting penetration of the BBB; however, hydrogel’s viscosity needs to be studied attentively, and surface treatment needs to be done to increase the adhesion between the PDMS and the hydrogel.^136,184,211^ Despite that, both models improve intercellular communications, and recent papers report employing integrated electrodes in devices to measure TEER, obtaining more reproducible and practical values.^212^

This layout with micropillars has been reported recently in several applications, and a summary of the recent publications is shown in Table 4. For example, Shi et al. (2023) constructed a BBB-glioma microfluidic chip to study antiglioma drug permeability through the BBB. They showed that the drug’s permeability coefficients in the microdevice were closer to the in vivo data obtained of traditional Transwell assay; however, the effect of the drugs on cancer cells was significantly lower than 3D cultured glioma cells due to the BBB. In conclusion, they demonstrated the necessity to consider the BBB while developing new antiglioma drugs.^33^

**Table 4:** Recent Publications of BBB-on-a-Chip with a Planar Design and Horizontal Configuration

		material		BBB cells		BBB characterization (approximate values)		
fabrication methods	chip design	chip	hydrogel	blood channel	brain channel	TEER (Ω cm^2^)	permeability (cm s^–1^)	ref
molding (soft lithography)	hydrogel barrier	PDMS	fibrin and Matrigel	hBMECs, PRCs and ACs (in fibrin)	NPCs (in Matrigel)	N/A	(×10^–7^)	^217^
							Texas Red–dextran (40 kDa):	
							•hBMECs, PRCs and ACs: 1.8	
							•hBMECs, PRCs, ACs, NPCs: 1	
molding (soft lithography)	micropillars	PDMS	Geltrex	hBMECs	hMC3 and hACs (in Geltrex)	17.83	(×10^–4^)	^218^
							fluorescent dextran (70 kDa):	
							•control: 60	
							•BBB: 10	
commercial chip (OrganoPlate, Mimetas)	micropillars (phase guide) with hydrogel barrier	low-absorbing material (n/a PDMS)	collagen type I	TY10	hPRCs and hACs (in collagen I)	YT10:9	(×10^–6^)	^45^
						TY10 + hPRCs: 18	FITC-dextran (20 kDa):	
							•YT10: 6–7	
							•YT10 + hPRCs: 0.6–0.7	
commercial chip (OrganoPlate, Mimetas)	micropillars (phase guide) with hydrogel barrier	low-absorbing material (n/a PDMS)	collagen type I	HUVECs	ECM (collagen I) without cells	N/A	(×10^–3^)	^32^
							FITC-dextran (40 kDa):	
							•control (without HUVECs): 2.01	
							•HUVEC: 6.72	
							•HUVEC, 10 μM Aβ: 3.52	
							•HUVEC, 20 μM Aβ: 5.62	
							•HUVEC, 30 μM Aβ: 5.89	
molding (soft lithography)	micropillars	PDMS	fibrin and Matrigel	hBMECs and hBVP	hACs (in fibrin) and glioma U251 (in Matrigel)	control (transwell): 45	(×10^–6^)	^33^
							FITC-dextran:	
							•4 kDa: 2.25	
							•40 kDa: 0.532	
							•70 kDa: 0.387	
molding (mold made with laminate material by laser cutter)	multilevel channels (hydrogel barrier)	PDMS	fibrin	iPS-ECs, hPRCs, hACs and GBM cell line 22	N/A	N/A	(×10^–6^)	^108^
							FITC-dextran:	
							•4 kDa: 0.032	
							•10 kDa: 0.032	
							•40 kDa: 0.05	
							nanoparticles:	
							•Bare NP: 0.04	
							•pPLD NP: 0.03	
							•AP2 NP: 0.02	
molding (soft lithography–microdevice) and molding (3D-printed mold–macro device)	micropillars associated with multilevel channels	PDMS	fibrin	iECs	iECs, ACs, and PRCs (in fibrin)	N/A	(×10^–6^)	^137^
							FITC-dextran:	
							•10 kDa: 0.17	
							•40 kDa: 0.042	
molding (3D-printed mold–macro device)	micropillars associated with multilevel channels	PDMS	Cultrex (Trevigen)	BMECs, PRCs, MB-231, MB-231Br	iPSC-derived NPCs, ACs and HMC3 (in Cultrex)	370	(×10^–6^)	^136^
							FITC-dextran	
							•4 kDa: 0.6	
							•70 kDa: 0.08	
molding (soft lithography)	micropillars	PDMS	fibrin	iECs, hPRCs and hACs	hPRCs, hACs, MDA-MB-231, MDA-LM2-4175, and MDA-BrM2a	N/A	(×10^–6^)	^117^
							FITC-dextran (40 kDa):	
							•iECs: 0.62	
							•iECs + hPC: 0.62	
							•iECs + hAC: 0.5	
							•iECs + hPC + hAC: 0.32	
molding (soft lithography)	micropillars	PDMS	hyaluronic acid–collagen I conjugated	hCMEC/D3, and HBVP	NSC	N/A	FITC-dextran (70 kDa):	^119^
							•NSC + ECs: 2.41	
							•NSC + ECs + PCs: 0.656	
molding (soft lithography)	micropillars	PDMS	fibrin	hBMEC and hPRCs or hBM-MSC	hLFs and hACs (in fibrin)	N/A	(×10^–6^)	^118^
							FITC-dextran (10 kDa):	
							•hBMECs + hPCs + hACs: 13.8	
							•hBMEC + hBM-MSC + hAC: 9.6	
molding (soft lithography)	micropillars	PDMS	fibrin and hyaluronic acid	hBMECs and hPC–PL	LFs and NHA (in fibrin)	N/A	(×10^–6^)	^194^
							FITC-dextran:	
							•10 kDa: 0.86	
							•70 kDa: 0.31	
molding (soft lithography)	micropillars/microcannels	PDMS	Cy5-labeled collagen I	hHA, hPC, and hCMEC/D3	GBM cell line U87	control (transwell): 30–40	(×10^–6^)	^215^
							FITC-dextran (10 kDa): 1.4	
							nitrofurantoin (238 Da): 3.8	

Brain metastases are a common occurrence in patients with cancer. However, the mechanisms of cancer cell movement across the BBB are poorly understood. In a recent study by Hajal et al. (2021), a 3D in vitro BBB model was used to show that ACs can promote cancer cell transmigration. This model overcomes the limitations of other available models by making it more relevant to human physiology and morphology. It also allows for identifying cellular and molecular factors that directly affect extravasation while increasing experimental throughput and spatiotemporal resolution.^117^ Both works have the use of fibrin in common. In models with coculture involving brain cells (ACs, microglia, or neurons), fibrin is commonly used due to its capacity to mimic soft tissue.^213^ The mechanical properties of this gel can be turned by adjusting the concentration, achieving a low mechanical modulus, which is favorable for neural cell scaffolding.^214^

Fungal brain infection was modeled in NVU with a functional BBB by Kim et al. (2021). They demonstrated the ability of , the fungal meningitis pathogen, to penetrate the BBB via coculture of stem cells, brain ECs, and brain PRCs on a microfluidic chip with micropillars. Furthermore, they found that the tight junctions were not altered when the pathogen, which forms cell clusters, penetrated the BBB, suggesting a transcytosis-mediated mechanism.^119^ Also, in BBB penetration, Peng et al. (2020) published surface modifications to improve the screening of molecules and nanoparticles. Instead of micropillars, they use microchannels to connect the blood and brain channels, acting as a physical barrier between the compartments. Moreover, using a photo-cross-linkable copolymer, they could coat and functionalize BBB chip, providing a covalent layer attached to extracellular matrix proteins, allowing the coculture and formation of a mimic of cerebral endothelium expressing tight junction markers. In addition, the BBB penetration NPs can target glioma cells cultured in the brain compartment of their chip, predicting the permeability of small molecules and nanovectors.^215^

Angiogenesis is a necessary process occurring in normal or pathological states, which arises in the formation of new blood vessels from pre-existing vessels.^216^ Many authors used horizontal design chips with micropillars to study these characteristics of cerebral angiogenesis. Lee et al. (2020) conducted a study to investigate the impact of PRCs and ACs on the architecture of ECs in a chip with micropillars (Figure 8B­(i)). Their research confirmed the importance of angiogenic triculture in achieving the phenotypes of BBB vasculature, such as maximized TJs protein expression and minimized vessel diameter, as shown in Figure 8B­(ii,iii). Additionally, they found that the triculture condition resulting in lower vascular permeability when compared to monoculture (Figure 8B­(iv)).^194^ Kim et al. (2021) provided evidence of the reparative effects of human bone marrow mesenchymal stem cells on BBB repair. They discovered that stem cells act as perivascular PRCs in the tight reformation of the BBB, with a greater capacity to constrict blood vessels than PRCs.^118^

Recently, the multilevel channel design was reported to be less than the micropillar design. However, these layouts are sometimes seen to be combined in the same microfluidic chip. Straehla et al. (2022) developed a microfluidic model of human glioblastoma to study the transportation of BBB-penetrating nanoparticles. They developed the hydrogel barrier with fibrin and reported a vascularized glioblastoma model with self-assembled ECs, ACs, and PRCs in coculture. They validated the platform’s ability to model in vivo BBB transport compared with transport across mouse brains. Also, the therapeutic potential of functionalized nanoparticles was investigated, and their efficacy improved.^108^ Lyu et al. (2021) reported a functional neurovascular unit on a microfluidic chip (Figure 8C­(i)) that recapitulates the function of the BBB as a neurophysiological model of ischemic stroke and as a clinically relevant model through the response of invading cells (MB-231 and MB-231Br) (Figure 8C­(ii)). They used a basement membrane extract as a hydrogel barrier (Cultrex, Trevigen) and demonstrated distinct neurorestorative effects for each type of stem cell.^136^ Hajal et al. (2022) described a protocol for device fabrication, device culture and downstream imaging, and protein and gene expression analysis for in vitro BBB self-assemblyd. This BBB model exhibited relevant cellular organization, morphological features, and molecular permeability within the expected range in vivo, compared to 2D assays.^137^

Regarding the fabrication method and microchip dimensions, it was observed that chips made by soft lithography reach sizes around 100–200 nm while being driven by micromolding using a 3D-printed mold, which is around 500 nm. The permeability in microchips with parallel design with horizontal configuration showed values around 10^–7^–10^–6^ cm s^–1^, with values close to those in vivo (∼10^–7^ cm s^–1^). Some authors reported that permeability decreases with coculture: using FITC-dextran 10 kDa, Kim et al. showed values around 10^–5^ cm s^–1^ for EC monoculture, compared with 10^–6^ cm s^–1^ for coculture (ECs with ACs and PRCs), as did Lee et al. with values around 10^–6^ cm s^–1^ for EC monocultures and 10^–7^ cm s^–1^ for coculture (Figure 8C­(iii)).^118,194^ Even on a larger device compared with soft lithography devices Straehla and collaborators reached values around 10^–7^ cm s^–1^ to the permeability of FITC-dextran (40 kDa) in the microchip and in the mouse brain.^108^

#### 4.2.3 Cylindrical Design

Most channels in a planar configuration are engineered with rectangular cross sections, which can result in irregular shear stress on the vascular endothelium. In this way, fabricating cylindrical channels (Figure 7C) can be a potential solution, benefiting from constant shear stress along the entire wall vessels.^219^ In addition, the cylindrical design allows full contact between the cells and is reported to mimic blood vessels, enabling omnidirectional communication between the ECs and another cell type.^220^ Using needles or wires as sacrificial molds, the channels with circular cross sections can be molded in ECM gel, in which the diameter of the channels can be turned, adjusting the mold size.^221^ Due to the cylindrical geometry of the vessels formed and the chip design, TEER measurements are challenging to achieve, but confocal microscopy enables the measurement of vascular permeability using these models.^137,222^

Different hydrogels from ECM can be used to mold these tube-like vessels, as summarized in Table 5. Seo et al. (2022) studied BBB-associated chemosensitivity and drug delivery on glioblastoma, testing three different drugs: temozolomide, vincristine, and doxorubicin. They produced a pump-free cylindrical microchip with microvessels molded in collagen I, as shown in Figure 8D­(i). They worked with cocultures of brain cells (ECs, PRCs, and ACs) and brain tumor cells (glioma cell lines). The study’s results demonstrate that their platform can examine the physiology of the BBB and monitor drug responses based on the interactions between brain tumors and the BBB (Figure 8D­(ii,iii)).^34^ Another pump-free BBB-on-a-chip model for understanding barrier properties and drug response was developed by Yu et al. (2020). Using collagen I to pattern the microchannel and cocultured ECs, ACs, and PRCs in this matrix, they mimic the 3D BBB structure. Also, they added tumor necrosis factor to recapitulate neuroinflammatory conditions, treating the BBB model with the glucocorticoid drug and observing the protection of BBB.^223^

**Table 5:** Recent Publications of BBB-on-a-Chip with a Cylindrical Design

	material					BBB characterization (approximate values)		
fabrication methods	chip	hydrogel	coating	sacrificial material	BBB cells	TEER (Ω cm^2^)	permeability (×10^–6^) (cm s^–1^)	ref
wire molding (hydrogel) molding (PDMS chip)	PDMS	collagen I	N/A	microneedles	HBVP, HA, HBMEC, T98G and U87MG	N/A	FITC-dextran:	^34^
				diameters: 550 and 235 μm			•4 kDa: 0.0254	
							•40 kDa: 0.0183	
							fluorescein salt:	
							•376 Da: 1.23	
wire molding (hydrogel) molding (PDMS chip)	PDMS	collagen I and Matrigel	collagen IV and fibronectin	nitinol wire	iBMECs from iPSCs (HD180 and HD-corrected iPSCs were confirmed)	control (transwell):	Lucifer yellow (444 Da):	^225^
				diameter: 150 μm		•HD180: 1024	•transwell: 2 (day 2)	
						•HD corrected: 2067	•transwell: 4 (day 10)	
							•chip (HD180 and HD-corrected): 0.1	
wire molding (hydrogel) molding (PDMS chip)	PDMS	collagen I and Matrigel	collagen IV and fibronectin	nitinol wire	iBMECs, iECs and iPRCs.	N/A	Lucifer yellow (444 Da):	^129^
				diameter: 150 μm			•transwell (iBMECs): 1	
							•transwell (iECs): 0.1	
							•chip (iBMECs): 100	
							•chip (iECs): 10	
wire molding (hydrogel) molding (PDMS chip)	PDMS	collagen I and Matrigel	collagen IV and fibronectin	nitinol wire	iBMECs	N/A	N/A	^130^
				diameter: 150 μm				
molding (soft lithography)	PDMS	collagen I	N/A	diameter: 300–400 μm (SU-8 molds)	ECs, PRCs, and ACs (neonatal rat brain cells)	control (transwell):	N/A	^223^
						•day 1: 105		
						•day 3: 160		
						•day 9: 180		
						chip:		
						•day 1: 190		
						•day 3: 300		
						•day 9: 380		
molding (soft lithography)	PDMS	collagen I cross-linked with genipin	N/A	acupuncture needle	hBECs (TY10 cell Line)	N/A	FITC-dextran (10 kDa):	^224^
				diameter: 100 μm			•transwell: 4	
							•chip: 2	
wire molding (hydrogel) molding (PDMS chip)	PDMS	collagen I and Matrigel^Ⓡ^	N/A	nitinol wire	iBMECs from iPSCs, and HUVECs.	control (transwell): 2500	N/A	^128^
				diameter: 150 μm				

Using soft lithography and collagen I, Salman et al. (2020) designed and validated a BBB microfluidic model to enable advanced optical imaging. They utilized a brain microvascular ECs model system in vitro that was amenable to multiple high-resolution imaging modalities, including transmission electron microscopy, spinning disk confocal microscopy, and advanced lattice light sheet microscopy. In addition, the barrier function was validated by measuring the permeability of fluorescent dextran and human monoclonal antibodies.^224^ Linville’s work (2020) showed the influence of ECM components on dhMEC angiogenesis, in which collagen I, collagen I + fibrin, collagen I + Matrigel, and collagen I + fibronectin were tested, showing that Matrigel supplementation increased sprouting compared to fibronectin and fibrin. The model was developed using collagen I + Matrigel with two channels separated by 100–200 μm, providing a tool for studying physiological and pathological brain angiogenesis.^128^

This cylindrical model was also used to study gene expression as a function of the BBB in vitro with an isogenic family of fluorescently labeled iBMECs and brain pericyte-like cells (iPRCs). The microvessel was constructed with collagen I supplemented with Matrigel and coated with collagen IV and fibronectin, showing that ECs cocultured with PRCs in a 3D microenvironment enhance endothelial identity and BBB phenotype, leading to altered cytokine and angiogenic responses. The study analyzed the response of iBMEC microvessels to chemical injuries. Two chemicals, namely, menadione and melittin, were used for this purpose. Menadione caused delamination of the endothelium, leading to the partial collapse of the microvessel. On the other hand, melittin induced cell loss from the endothelium, thereby increasing the permeability of dextran (10 kDa).^129^ Linville et al. (2022) used this developed microdevice to understand how BBB dysfunction contributes to the progression of Huntington’s disease.^225^

The permeability measured with 10 kDa FITC-Dextran showed values around 10^7^–10^–6^ cm s^–1^ to different authors: Salman and collaborators observed values of 10^–6^ cm s^–1^ while Linville et al. foung values around 10^–7^ cm s^–1^.^129,224^ Seo et al. observed different values of FITC-dextran (4 kDa) permeability according to the complexity of the model, in which a monoculture of EC reached values of 10^–6^ cm s^–1^ and coculture 10^–7^ cm s^–1^ (Figure 8D­(iv)).^34^ Despite the difficulty in TEER measurements due to the small sizes of microfluidic chips, Yu and collaborators used the EVOM2 TEER instrument (from World Precision Instruments) to measure the voltage level in the lumen of the BBB and outside of the EC layer, reaching values around 200 Ω cm^2^,^223^ lower values when compared with the BBB in vivo (1500–8000 Ω cm^2^).^226^

## 5 Conclusions

OoC technology has rapidly advanced by combining cell culture with microfluidic technologies to model various tissues, including the lung, heart, kidney, liver, gut, and blood–brain barrier, in which the last one represents about 8% of OoC publications since 2010.^227^ BBB-OoC publications over the last 5 years (from 2020 to February 2025), have demonstrated growth in this research area, with an increase of 178% in the number of publications. Currently, several companies are working on commercializing different chips for use in OoC applications. However, these chips are often multipurpose and designed for various uses, which can compromise their accuracy in effectively mimicking the BBB. Moreover, there is a pressing need to create models beyond cellular organization, integrating sensors that can measure and monitor control parameters in real-time. Evidence of the lack of technological maturity in BBB model commercialization is reflected in the low percentage of BBB-related papers (around 8%) in collaborative publications over the past five years (Table 2). Despite this, the growing number of BBB publications in recent years indicates an increasing interest in and ongoing progress in this field.

Despite the reported advances in the microfluidics and BBB fields, there are still some challenges around microfabrication and operation. Concerning microfabrication, the choice of fabrication technique must consider aspects such as low cost, rapid prototyping, high resolution, and scalability.^228^ However, none of the techniques presented in this review to construct BBB models present all of these aspects. Also, the material selection to fabricate microfluidic devices must consider biocompatibility and adhesion properties.^13,229^ For instance, PDMS, the usual material chosen, is a very hydrophobic polymer, and this requires surface treatments and coatings to allow the ECM hydrogel or cell adhesion.^184,230^ In addition, since the PDMS may absorb hydrophobic small molecules (such as drugs and antibodies), the use of these chips without any pretreatments can affect the expected results, providing false positive/negative results.^231^

Regarding operating challenges, advanced imaging techniques (such as confocal and fluorescence microscopy) may be required to evaluate dynamic processes and cell behavior, which makes the use of these chips often unfeasible and restricted.^13,25^ Also, to create a dynamic system, connecting these devices to peristaltic or syringe pumps could be challenging due to these instruments’ compatibility with channels with micrometric dimensions.^25^ In addition, depending on the design configuration, the analysis tools for BBB characterization (i.e., TEER and permeability measurements) may not be easily integrated into the chips.^26^ Due to the interdisciplinarity involved in constructing the OoCs, collaborative efforts across different scientific areas are crucial for refining the existent BBB-OoC designs, making these chips accessible and easy to handle and monitor.

In spite of challenges in constructing and validating BBB models, their application to mimic complex diseases and screening purposes remains demanding. Just as there has been great growth in this area since the development of the first OoC to the present day, there is expected to be an equivalent leap of development in the next decade, overcoming the current challenges and translocating these models from the laboratory scale to the market.

## 6 Future Perspectives

Efforts are still necessary to mimic the BBB and obtain an easy microphysiological system that can be used in the biology and medical fields. Future trends in the development of BBB-on-a-chip technology highlight three main areas of focus: (i) real-time monitoring and characterization, (ii) cellular aspects, and (iii) the development of cellular microenvironments. From a monitoring standpoint, the evolution of these devices is trending toward greater integration of elements that allow for real-time assessment of critical physical parameters (such as electrical sensors measuring TEER), as well as various chemical and biochemical signals.^232,233^ Consequently, we anticipate the creation of modular networks that combine microfluidic chips with biosensor systems. Furthermore, a range of imaging techniques beyond approaches based on light propagation (brightfield and phase contrast) or fluorescence detection are available, such as approaches based on ionizing radiation, magnetic fields, or ultrasound.^205^ Optical second harmonic generation, for example, is a nonlinear optical process that can be used for cell membrane monitoring.^234,235^ Another technology for noninvasive cell analysis is Raman microspectroscopy, which enables label-free identification of metabolic changes in cells with high sensitivity and has been successfully applied to both 2D and 3D cell cultures.^236,237^ The available alternatives differ in complexity, cost, resolution, image quality, and application scope. Therefore, the most suitable technological approach must be chosen based on the analyzed system’s characteristics and the relevant information required.

From the cellular perspective point, combining organoids with OoC technology offers a promising approach to enhance our understanding of BBB development, function, and diseases.^238^ Organoids, which are 3D structures derived from pluripotent stem cells or specific progenitor cells, may provide several advantages, including cellular diversity, self-organization, and physiological responses that closely resemble native tissues.^239,240^ Additionally, induced pluripotent stem cells (iPSCs) can be sourced from any individual, allowing researchers to model interindividual variability. This capability is a valuable tool for understanding disease mechanisms and developing personalized therapies tailored to patient-specific genotypes and phenotypes.^241^ In this context, although few uses of organoids in BBB-on-a-chip models have been reported, we visualize the trend of integrating organoids into OoC technologies in the future, as has been highlighted in other recent reviews.^242^ However, it is important to note that using organoids also comes with limitations, particularly the lack of biochemical and biomechanical stimuli from the surrounding tissue microenvironment and the absence of functional vascular structures.^243^

From a microenvironmental perspective, selecting the appropriate hydrogel for BBB devices is crucial. The biomaterial must not only fulfill biological requirements by providing a favorable environment for the cultivation of cells or organoids but also possess adequate mechanical strength to support the integration of these perfusion chips, such as syringe pumps, peristaltic pumps, or pumpless tilting plane systems. This is important because shear stress serves as a key stimulus for the growth of endothelial cells and the expression of adherens and tight junction proteins.^160,244^ Materials found in the extracellular matrix, such as collagen and fibrin, are commonly used in BBB models, along with commercial basement membrane extracts like Matrigel^Ⓡ^ and Geltrex^Ⓡ^.^245^ However, the high cost and poor mechanical properties of these materials highlight the necessity to develop new biomaterials that can accurately replicate this microenvironment. Creating synthetic matrices that replicate the structural sophistication, biochemical complexity, and dynamic functionality of native tissues remains a significant challenge, especially regarding their integration into engineered systems. Most synthetic hydrogels are bioinert, which means they do not naturally interact well with cells.^246,247^ To improve cell–scaffold interactions, specific bioactive peptides and proteins can be immobilized on the hydrogel surfaces, such as in the use of hydrogels made from adhesive polyethylene glycol (PEG) functionalized with RGD peptides, which have shown enhancements in cell survival, spreading, migration, and specialized cell functions.^248,249^ Despite the growing use of bioactive synthetic matrices in recent years, their application in constructing BBB-on-a-chip models has not yet been reported.

The widespread adoption of the OoC models relies heavily on ongoing efforts to minimize costs and improve the ease of operation and monitoring of existing models. In the case of the BBB, the scientific community needs to engage in discussions about which essential parameters genuinely define existing models as barrier models. Standardizing these parameters would provide valuable guidance for researchers in engineering and related fields, helping them achieve long-term development goals.

## Supplementary Material


