# Supplementary figures and images for: Microfluidic Systems to Mimic the Blood–Brain Barrier: from Market to Engineering Challenges and Perspectives
Source: ACS Biomater Sci Eng. 2025 Jun 25;11(7):3789–815. doi: 10.1021/acsbiomaterials.4c02221 (PMC12264779; doi:10.1021/acsbiomaterials.4c02221)

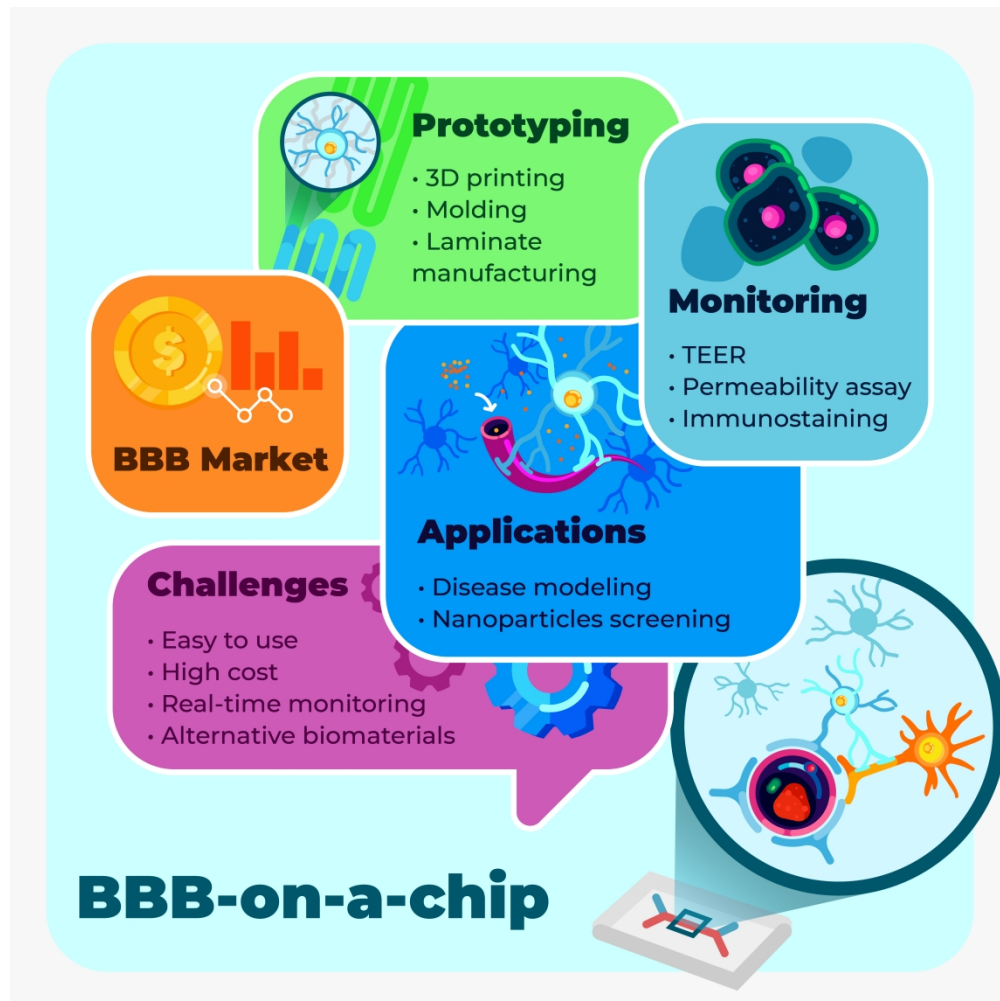

381x381mm (300 x 300 DPI)

Supplement: Supplementary file 1 [file ab4c02221_si_001.pdf]
